# Surfactants tailored by the class *Actinobacteria*

**DOI:** 10.3389/fmicb.2015.00212

**Published:** 2015-03-19

**Authors:** Johannes H. Kügler, Marilize Le Roes-Hill, Christoph Syldatk, Rudolf Hausmann

**Affiliations:** ^1^Technical Biology, Institute of Process Engineering in Life Sciences, Karlsruhe Institute of TechnologyKarlsruhe, Germany; ^2^Biocatalysis and Technical Biology Research Group, Institute of Biomedical and Microbial Biotechnology, Cape Peninsula University of TechnologyBellville, South Africa; ^3^Bioprocess Engineering, Institute of Food Science and Biotechnology, University of HohenheimStuttgart, Germany

**Keywords:** biosurfactant, emulsifier, glycolipid, lipopeptide, trehalose lipid, *Rhodococcus*, rhamnolipid

## Abstract

Globally the change towards the establishment of a bio-based economy has resulted in an increased need for bio-based applications. This, in turn, has served as a driving force for the discovery and application of novel biosurfactants. The class *Actinobacteria* represents a vast group of microorganisms with the ability to produce a diverse range of secondary metabolites, including surfactants. Understanding the extensive nature of the biosurfactants produced by actinobacterial strains can assist in finding novel biosurfactants with new potential applications. This review therefore presents a comprehensive overview of the knowledge available on actinobacterial surfactants, the chemical structures that have been completely or partly elucidated, as well as the identity of the biosurfactant-producing strains. Producer strains of not yet elucidated compounds are discussed, as well as the original habitats of all the producer strains, which seems to indicate that biosurfactant production is environmentally driven. Methodology applied in the isolation, purification and structural elucidation of the different types of surface active compounds, as well as surfactant activity tests, are also discussed. Overall, actinobacterial surfactants can be summarized to include the dominantly occurring trehalose-comprising surfactants, other non-trehalose containing glycolipids, lipopeptides and the more rare actinobacterial surfactants. The lack of structural information on a large proportion of actinobacterial surfactants should be considered as a driving force to further explore the abundance and diversity of these compounds. This would allow for a better understanding of actinobacterial surface active compounds and their potential for biotechnological application.

## Microbial surfactants and their applications

Microbially derived compounds that share hydrophilic and hydrophobic moieties, and that are surface active, are commonly referred to as biosurfactants. Many have been detected and described, and the majorityare molecules of low molecular weight. Within this group of low molecular weight microbial surfactants, the classes of lipopeptides or glycolipids, where fatty acid or hydroxy fatty acid chains are linked to either peptides or carbohydrates, have been extensively studied (Hausmann and Syldatk, 2014). The combinations of different types of hydrophilic and hydrophobic moieties within surfactants are innumerable and highly biodiverse. Due to their amphiphillic structures, surfactants act as emulsifying agents, resulting in low surface tensions of interphases. Often, microorganisms produce them when growing on hydrophobic carbon sources or when exposed to growth limiting conditions. It is hypothesized, that biosurfactants play a role in the uptake of various hydrophobic carbon sources thus making nutrients bioavailable, as well as the protection of bacteria from harsh environmental conditions (Ristau and Wagner, 1983; Vollbrecht et al., 1998; Philp et al., 2002). Some biosurfactants show antimicrobial effects and the distinction of secondary metabolites as antibiotics or biosurfactants is often not strict.

Biosurfactants, compared to chemically derived surfactants, are independent of mineral oil as a feedstock, they are readily biodegradable and can be produced at low temperatures. Furthermore, they are described to be less toxic, effective at low concentrations and show effects in bioremediation. Industrial interest in biosurfactants is not solely based on the bio-acitivity of these molecules, but is also due to the broader ecological awareness linked to their application, which in turn is driven by sustainability initiatives and green agendas (Marchant and Banat, 2012). Biosurfactants can be applied in various areas such as the nutrient-, cosmetic-, textile-, varnish-, pharmaceutical-, mining-, and oil recovery industries (Henkel et al., 2012; Marchant and Banat, 2012; Müller et al., 2012).

An example of an actinobacterial biosurfactant that has already entered the market and found industrial application, is the lipopeptide antibiotic daptomycin. This antibiotic is used in the treatment of diseases caused by Gram positive pathogens and has been marketed as Cubicin® by Cubist Pharmaceuticals. Other promising studies for the potential application of actinobacterial biosurfactants are in environmental applications such as bioremediation: Oil spills were successfully dispersed by biosurfactants produced by a *Gordonia* sp. (Saeki et al., 2009), a *Dietzia* sp. (Wang et al., 2014) and a *Rhodococcus* sp. (Kuyukina and Ivshina, 2010); and trehalose lipids were applied in microbial enhanced oil recovery and the cleaning of oil storage tanks (Franzetti et al., 2010). In medical applications, the production of biosurfactants are generally considered safer than synthetically produced compounds due to high enzymatic precision during synthesis. Antiproliferation activities of cancerogenic cells could be induced by application of various glycolipids (Isoda et al., 1997; Sudo et al., 2000). In cosmetic applications, the use of trehalose lipids is favored above that of sodium dodecyl sulfate as it causes less irritation (Marques et al., 2009).

**Figure 1:**
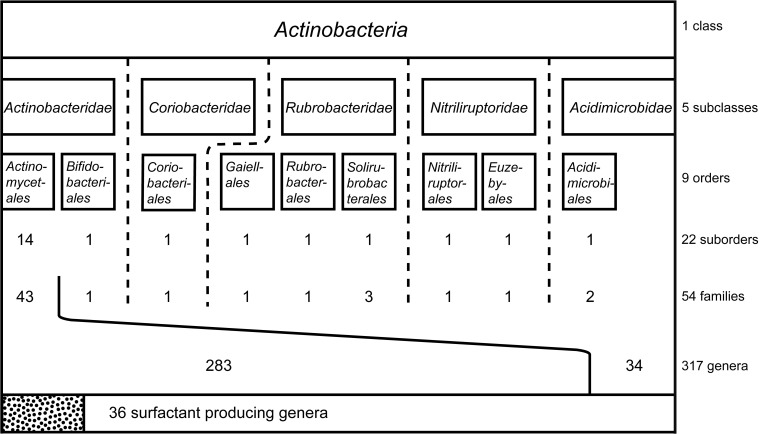
**Systematic classification of the class *Actinobacteria* including subclasses and orders**. Suborder, families and genera examined for the production of biosurfactants and bioemulsifying compounds are displayed in numbers. Thirty six surfactant-producing genera are reported, all belonging to the largest order within the *Actinobacteria*: *Actinomycetales*.

Different types of biosurfactants or bioemulsifiers have been described to be produced as secondary metabolites within the class *Actinobacteria*, and to the best of our knowledge, all of the producing species belong to the order *Actinomycetales* (Figure 1). The following section of the review will focus on the different types of actinobacterial biosurfactants reported in literature as well as their key structural features and bio-activities.

## Metabolite production within the class *Actinobacteria*

Over the past few decades, there has been an increased interest in the discovery of bioactive metabolites with novel bioactive properties and their potential for application in medical- or industrial-based processes. Microbial products are still considered to be the most promising source for the discovery of novel chemicals or therapeutic agents (Berdy, 2005). In addition, vast microbial genetic resources remains untapped and can lead to the development of novel bioactive metabolites.

In contrast to primary metabolites, secondary metabolites often accumulate and have miscellaneous chemical compositions that are species-specific. These secondary metabolites often exhibit bioactivity and are therefore of great interest to various industries. The most dominant source of microbially derived bioactive compounds is a group of bacteria known to have relatively large genomes and constitutes one of the main phyla within the *Prokaryotes*: The class *Actinobacteria* (Ludwig and Klenk, 2001). The class *Actinobacteria* play important roles in the environment, e.g., nutrient cycling, but also include major plant, animal and human pathogens (Embley and Stackebrandt, 1994), well known examples are the causative agents of leprosy and tuberculosis. Baltz (2008) assumed 5–10% of their genome coding capacity to be used for the production of secondary metabolites and indeed more than 35% of all known bioactive microbial metabolites and more than 63% of all known prokaryotic bioactive metabolites arise from actinobacteria (Bérdy, 2012). Most secondary metabolite producers described belong to families of the *Actinomycetales*, but it is estimated that only ~1% of them are culturable (Bérdy, 2012). Many of these actinobacterial secondary metabolites exhibit antibacterial, antifungal, antitumor, anticancer and/or cytotoxic properties (Manivasagan et al., 2013). Antibiotics, with around 10,000 compounds described (Bérdy, 2012) is by far the largest group of metabolites isolated from actinobacteria. Depending on their chemical nature, the huge number of antibiotic compounds can roughly be classified into *peptides*, *aminoglycosides*, *polyketides*, *alkaloids*, fatty acids, and *terpenes* (Manivasagan et al., 2013; Abdelmohsen et al., 2014). Besides antibiotics, other actinobacterial compounds described are bioactive compounds with pharmacological activity (pheromones, toxins, enzyme inhibitors, receptors and immunological modulators), with agricultural activity (pesticides, herbicides and insecticides) and other industrially relevant properties (pigments and surfactants). Most compounds are derived from members of the genus *Streptomyces*, however, other so-called “rare” actinomycetes are increasingly playing a more important role in the production of biocompounds (Berdy, 2005; Kurtboke, 2010).

To fully understand the taxonomic distribution of the actinobacterial strains identified to produce biosurfactants and bioemulsifying compounds, taxonomic data of the class *Actinobacteria* was evaluated. Information were retrieved from the taxonomy browser of the National Center for Biotechnology Information^1^ considering 16S rRNA gene sequence based reclassifications according to Zhi et al. (2009) and Goodfellow and Fiedler (2010). The order *Thermoleophilales* that has been reclassified into a new class (Euzéby, 2013) has been excluded and the recently identified order *Gaiellales* has been included (Euzéby, 2012). Overall, the class *Actinobacteria* contains five subclasses and nine orders with a total of 54 families (Figure 1). The largest order, *Actinomycetales*, is divided into 14 suborders and contains by far the highest diversity within the class *Actinobacteria*. It is therefore not surprising that biosurfactants reported in literature focuses on members of this order. The next few paragraphs will go into more detail around the different types of biosurfactants that have been identified to be produced by actinobacterial strains, their production, purification and structural elucidation, as well as the clear influence of the environment the producer organism is found in and their ability to produce biosurfactants.

## Trehalose-comprising glycolipids

The best described biosurfactants amongst the actinobacteria are glucose-based glycolipids, most of which have a hydrophilic backbone consisting of two α,α-1,1 glycosidic linked glucose units forming a trehalose moiety. Different types of trehalose-containing glycolipids and their producers have been extensively reviewed (Asselineau and Asselineau, 1978; Asselineau and Lanéelle, 1998; Franzetti et al., 2010; Kuyukina and Ivshina, 2010; Shao, 2011; Khan et al., 2012). Those of the class *Actinobacteria* are mainly found within the genera *Rhodococcus, Mycobacterium*, *Nocardia, Arthrobacter* and *Corynebacterium*, and less frequently within the genera *Tsukamurella, Brevibacterium*, and *Micrococcus* (Tables 1, 2). Different structures of trehalose lipid comprising amphiphilic molecules have been reported: Acyl chains with glycosidic linkages to glucose or trehalose units have been reported to vary in number of occurrence, length and type, as well as the position (and number) of their linkage to the sugar rings and exhibit different cellular functions.

**Table 1:** **Mycolic and corynemycolic containing trehalose lipids that are of actinobacterial origin**.

**Species**	**Strain**	**TL mycolic acid ester**	**References**
*Arthrobacter paraffineus*	KY 4303	TL mycolic (C32–C36)	Suzuki et al., 1969
*Brevibacterium* sp.	KY 4304/4305	TL mycolic (C32–36)	Suzuki et al., 1969
*Brevibacterium vitarumen*	12143	TL dimycolic (C28–C38)	Lanéelle and Asselineau, 1977
*Corynebacterium diphtheriae*	n.a.	Glucose mycolic (C32)	Brennan et al., 1970
*Corynebacterium* spp. *(fasciens, pseudodiphtheriae)*	KY 3543 KY 3541	TL mycolic (C32–36)	Suzuki et al., 1969
*Corynebacterium matruchotii*	ATCC 14266	TL dimycolic (C28–C38)	Datta and Takayama, 1993
*Mycobacterium* spp. *(smegmatis, tuberculosis)*	BCG, n.a.	Glucose mycolic (C32)	Brennan et al., 1970
*Mycobacterium* spp.^*^ (*bovis, fortuitum, kansaii, malmoense, phlei, tuberculosis, smegmatis, szulgai*, etc.)	Various	TL mycolic, dimycolic,	Reviewed in: Asselineau and Asselineau, 1978; Gautier et al., 1992; Asselineau and Lanéelle, 1998; Vergne and Daffé, 1998; Dembitsky, 2004; Ishikawa et al., 2009; Shao, 2011
*Nocardia* spp.	n.a.	TL mycolic (C32–36)	Suzuki et al., 1969
*Rhodococcus* spp.^*^ (*erythropolis, opacus, ruber*, etc.)	Various	TL mycolic,dimycolic,	Reviewed in: Asselineau and Asselineau, 1978; Lang and Philp, 1998; Kuyukina and Ivshina, 2010; Shao, 2011; Khan et al., 2012
**EXAMPLES OF MYCOLIC ACID CONTAINING TREHALOSE LIPIDS**			
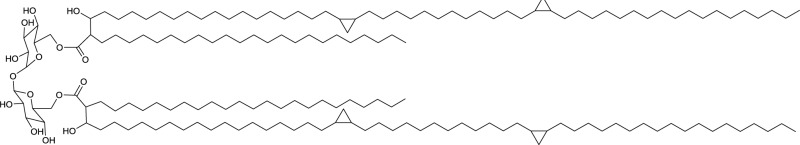			
**1**			
Trehalose dimycolate produced by *Mycobacterium tuberculosis*			
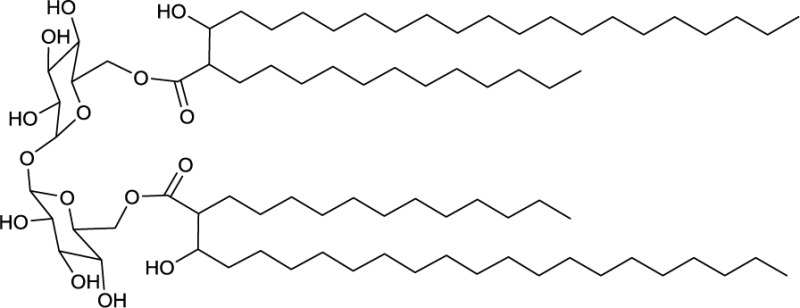
**2**			
Trehalose dicorynemycolate produced by *Rhodococcus erythropolis*			

* *Several producing species are reported; TL, trehalose lipid; n.a., information not available*.

**Table 2:** **Trehalose lipid ester of actinobacterial origin**.

**Species**	**Strain**	**TL ester**	**References**
*Arthrobacter* sp.	EK 1	TL tetraester (C12–C18)	Passeri et al., 1990
*Brevibacterium thiogenitalis*	No. 653	Glucose diester (C18)	Okazaki et al., 1969
*Micrococcus luteus*	BN56	TL tetraester (C9–C14)	Tuleva et al., 2009
*Mycobacterium* spp.^*^ (*africanum, bovis, fortuitum, tuberculosis*, etc.)	Various	TL ester	Reviewed in: Vergne and Daffé, 1998; Dembitsky, 2004; Shao, 2011
*Mycobacterium tuberculosis*	H37Rv	TL sulfolipid	Goren, 1970; Gilleron et al., 2004
*Nocardia farcinica*	BN26	TL succinic tetraester (C7-12)	Christova et al., 2014
*Rhodococcus* spp.^*^ (*erythropolis, longus, wratislavensis*, etc.)	Various	TL ester, TL succinic ester	Reviewed in: Asselineau and Asselineau, 1978; Lang and Philp, 1998; Kuyukina and Ivshina, 2010; Shao, 2011; Khan et al., 2012
*Tsukamurella pulmonis*	PCM 2578T	TL diester (C18–20/C4–5)	Pasciak et al., 2010a
*Tsukamurella spumae Tsukamurella pseudospumae*	DSM 44113, DSM 44114 DSM 44117	TL diester (C16–18/C4–6)	Kügler et al., 2014
*Tsukamurella tyrosinosolvens*	DSM 44370	TL diester (C16–18/C2–6)	Vollbrecht et al., 1998
**EXAMPLES OF TREHALOSE LIPID ESTERS**			
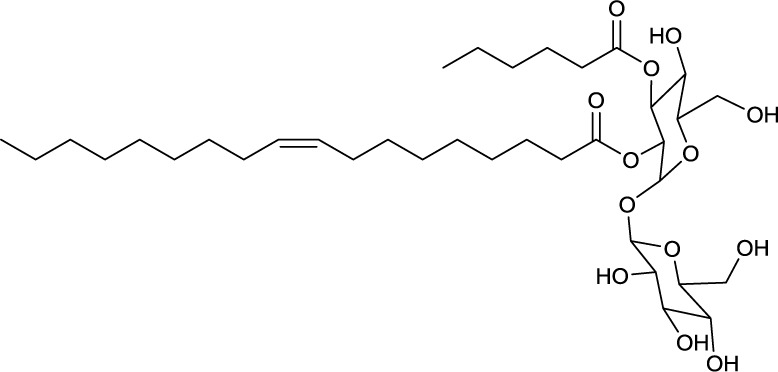		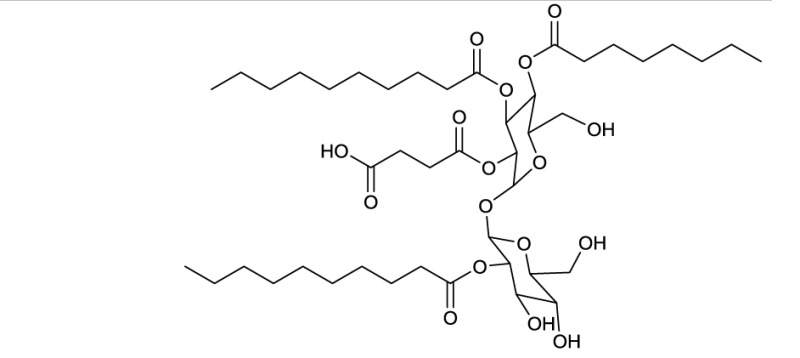	
**3**		**4**	
Trehalose diester produced by *Tsukamurella spumae*		Succinic trehalose tetraester produced by *Nocardia farcinia*	
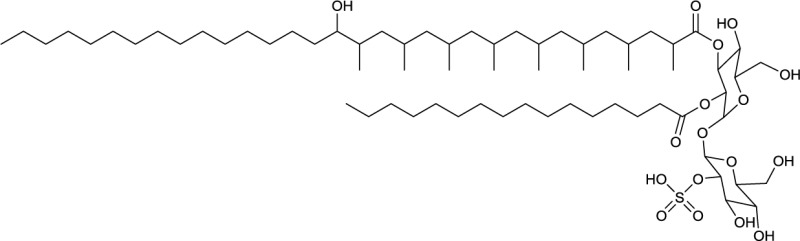			
**5**			
Diacetylated trehalose sulfolipid produced by *Mycobacterium tuberculosis*			

* *Several producing species are reported; TL, trehalose lipid*.

For the hydrophobic moiety of trehalose-comprising glycolipids, the structures of two main types of trehalose lipids have been elucidated: those carrying a mycolic fatty acid ester and those carrying a fatty acid ester.

The smallest hydrophilic backbone in glycolipids constitutes glucose, the building block of the sugar dimer trehalose. Complete structures of acylglucoses carrying mycolic acid esters have been elucidated and reported to be produced by isolates belonging to the genera *Corynebacterium* and *Mycobacterium* (Brennan et al., 1970) (Table 1), whereas acylglucoses carrying fatty acid esters have been described for *Brevibacterium* spp. (Okazaki et al., 1969) (Table 2).

### Trehalose lipid mycolic acid esters

Mycolic acids are long-chain fatty acids and a major component of the cell wall in various actinobacteria. Species-dependent, its lengths varies from 22 to 92 carbon atoms; they possess long β-hydroxy-α-branched acyl chains, including cyclopropane patterns and oxygenic groups. The synthesis of mycolic acids includes condensation reactions, and they are also referred to as eumycolic acid, corynemycolic acid and nocardio-mycolic acid, depending on their presence in *Mycobacterium* spp., *Corynebacterium* spp., and *Nocardia* spp., respectively (Asselineau and Lanéelle, 1998).

Mycolic acid comprising trehalose lipids (Table 1) can be distinguished into two different types, the trehalose mycolic lipids and the trehalose corynemycolic lipids. These mycobacterial trehalose mycolates or dimycolates are by far the most hydrophobic glycolipids. Linked to C6 (and C6′) of the sugar rings, they vary among species in length and branching. They are shaped to form bilayers, implemented in the outer cell wall and usually not found on the bacterial cell surface (Vergne and Daffé, 1998). Trehalose dimycolates (**1**, Table 1), also referred to as “cord factor,” serve a particular function for the cell. They act as virulence factors and have immuno-modulating activity (Shao, 2011). They may further be important to maintain a hydrophobic cell wall of the organism hence facilitating the uptake of hydrophobic carbon sources. The other type, trehalose lipids containing corynemycolic acid also carry β-hydroxy-α-branched fatty acid moieties and have been described to occur within the genus *Rhodococcus* (**2**, Table 1), carrying 30–56 carbon atoms and within the genus *Corynebacterium*, carrying 22–36 carbon atoms. They are also described to occur in mycobacteria (Brennan et al., 1970) and found in trehalose lipids of *Brevibacterium vitarumen* (Lanéelle and Asselineau, 1977), *Arthrobacter paraffineus* and a *Nocardia* sp. (Suzuki et al., 1969). Corynemycolic acids are much shorter than their mycobacterial counterparts: they lack functional groups and are often unsaturated. Within virulent strains of mycobacteria, five different sulfonated forms of trehalose esters have been found, varying in their acylation pattern (Khan et al., 2012).

### Trehalose lipid esters

Actinobacterial trehalose lipid esters are mainly acylated at C6/C6′ or at C2/C3 and are summarized in Table 2. The amount of hydrophobic chains linked to the trehalose unit varies from one to four, forming trehalose mono-, di-, tri- and tetraesters, but also octaesters (Singer et al., 1990) (**3**, Table 2). The acyl chains varies in lengths from C8 to C20, show an unsaturated pattern or form short succinoyl acids, giving the trehalose lipid an anionic character (Lang and Philp, 1998; Tokumoto et al., 2009) (**4**, Table 2). They are reported to be linked to the chain length present in hydrophobic carbon source fed to the producing strain. These glycolipid-linked medium chain length fatty acids are found within the following actinobacterial genera: *Arthrobacter*, *Brevibacterium, Caseobacter, Micrococcus, Mycobacterium, Nocardia, Rhodococcus*, and *Tsukamurella* (Table 2).

An exception among the trehalose lipid esters described, is sulfolipid 1 (Goren, 1970) (**5**, Table 2), a sulfonated and acylated trehalose lipid carrying phtio- and hydroxyphtioceranic compartments. They are known to contribute to the pathogenesis and virulence of *Mycobacterium tuberculosis*, the causative agent of tuberculosis. Diacyltrehalose sulfate, the biosynthetic precursor for sulfolipid 1, has recently been isolated from *M. tuberculosis* (Domenech et al., 2004) and has been used as a target for T-cell mediated recognization and elimination of *M. tuberculosis* infected cells (Gilleron et al., 2004).

### Oligosaccharide lipids

A glycosylated backbone of trehalose is found in oligosaccharide lipids (Table 3) carrying two to five sugar units. Trisaccharide lipids that have been reported for the class *Actinobacteria* all differ with respect to the acylation pattern of the third glucose unit. One sugar of the 1-1′ linked di-glucose backbone is further linked to a third sugar unit at C2 in the hydrophilic moeity of oligosaccharides produced by *Mycobacterium leprae* (Brennan, 1989) and *Tsukamurella tyrosinosolvens* (Vollbrecht et al., 1998). The third sugar unit is linked at C3 in a terrestrial actinomycete reported by Esch et al. (1999) and at C4 in a *Rhodococcus* sp. (Konishi et al., 2014) (**6**, Table 3). They also differ with respect to their hydrophobic nature. The latter two are acylated at all three sugar units, both carrying a C6 fatty acid moiety at the third sugar unit and succinic acid at the first sugar unit. Something that is rather exceptional is the acylation pattern at the trehalose backbone that, in its hydrophobic moieties, carries at each unit an acyloxyacyl structure in the *O*-ester linkage to the carbohydrate where the 3-hydroxy C8 or C10 fatty acid moiety is further acylated with a C6 fatty acid (**6**, Table 3). The *Tsukamurella* sp. trisaccharide lipids are acylated at two sugar units, each carrying two ordinary C8–C10 fatty acid units. Furthermore, a tetrasaccharide lipid form of this glycolipid has also been found to occur (Vollbrecht et al., 1998) (**7**, Table 3).

**Table 3:** **Actinobacterial oligosaccharide lipids**.

**Species**	**Strain**	**Oligosaccharid lipids**	**References**
*Mycobacterium* spp.^*^ (avium, *kansaii, leprae, linda, malmoense, smegmatis, szulgai, tuberculosis*)	Various	oligosaccharide ester, phenolic glycolipids	Reviewed in: Saadat and Ballou, 1983; Brennan, 1989; Dembitsky, 2005b
*Nocardia corynebacteroides*	SM1	Pentasaccharide succinic octaester (C2–C8)	Powalla et al., 1989
*Rhodococcus* sp. *Rhodococcus fascians*	NBRC 1097287 NBRC 12155	Trisaccharid succinic tetraester (C8-*O*-C6/C6)	Konishi et al., 2014
*Tsukamurella tyrosinosolvens*	DSM 44370	Tri/tetrasaccharide ester (C8-10)	Vollbrecht et al., 1998
**EXAMPLES OF OLIGOSACCHARIDE LIPIDS**			
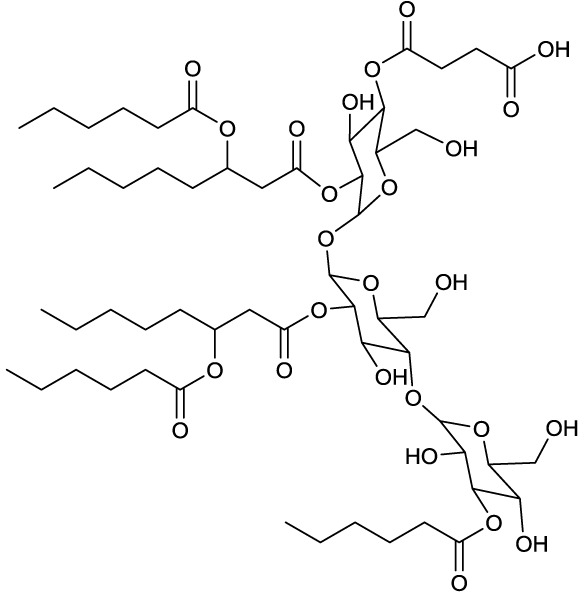		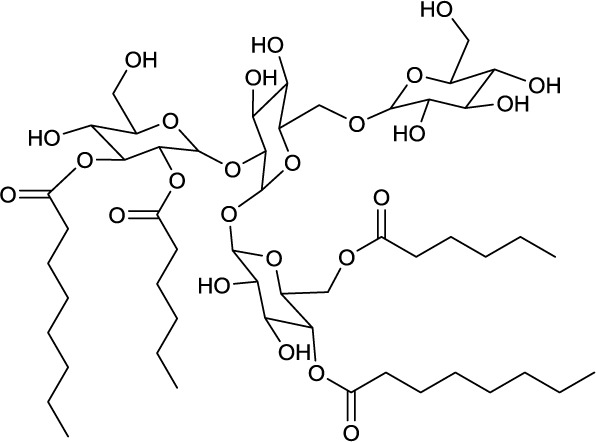	
**6**		**7**	
Succinic trisaccharide lipid produced by *Rhodococcus fascians*		Tetrasaccharide lipid produced by *Tsukamurella tyrosinosolvens*	
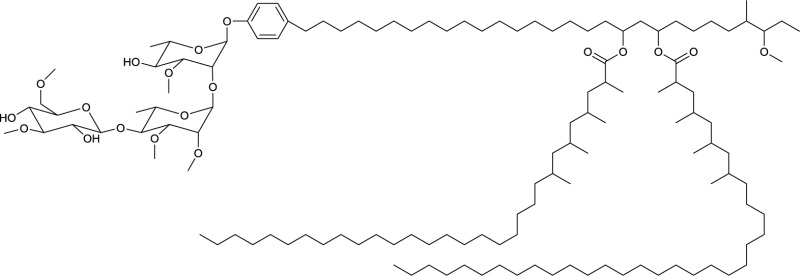			
**8**			
Methylated dirhamnose/glucose phenol phtiocerol named phenolic glycolipid I of *Mycobacterium leprae* Brennan, 1989			

* *Several producing strains are reported*.

Non-trehalose based oligosaccharide lipids are found within phenol-phtiocerol glycosides in various mycobacteria. These oligosaccharide lipids, also termed phenolic glycolipids, contain tri- and tetraglycosyl units composed of various methylated sugars that are mainly based on rhamnose and partly on fucose, glucose and arabinose (Brennan, 1989). The rarely described phenolic acylation pattern is bound to dimycocerosyl phtiocerol acyl groups. The phenolic glycolipid I of *M. leprae* carries three mycocerosyl acyl groups each in length of C30–C34 (Brennan, 1989) (**8**, Table 3).

In industrial and environmental processes the potential of trehalose lipids could become valuable as they have shown interesting properties in several studies that focus on the remediation of hydrocarbon contaminated soils, the removal of suspended solids from wastewater (Franzetti et al., 2010) and in enhanced oil recovery (Christofi and Ivshina, 2002). However, most research are centered around the bio-activity of trehalose lipid molecules that exhibit biomedical properties such as antimicrobial, antiviral (Azuma et al., 1987; Watanabe et al., 1999; Shao, 2011) and anti-tumor activities (Sudo et al., 2000; Franzetti et al., 2010; Gudiña et al., 2013). Due to their functions in cell membrane interactions they can act as therapeutic agents (Zaragoza et al., 2009; Shao, 2011) or have an impact on the pathogenesis of causative agents of infections, such as those caused by pathogenic *M. tuberculosis, Corynebacterium diphteriae*, and the opportunistic pathogens, *Mycobacterium avium, Mycobacterium intracellulare, Nocardia asteroides, Corynebacterium matruchotii*, and *Corynebacterium xerosis* (Kuyukina and Ivshina, 2010). Trehalose lipids can be excreted into the cultivation supernatant or can be produced as non-covalently linked lipids bound to the cell wall or they can be cell wall integrated thus posing limits to quantities produced by the organisms, a disadvantage for its potential exploitation in large scale production processes.

## Non-trehalose glyolipids

### Hexose-comprising glycolipids

Besides the trehalose-containing biosurfactants and its congeners, several glycolipids have been elucidated that are produced by actinobacteria and share other hydrophilic moieties. By simply varying the carbon source in the growth media from n-alkanes to either sucrose or fructose, the hydrophilic part of the surfactant produced was reported to be switched from trehalose to fructose by members of the genus *Arthrobacter*, *Corynebacterium*, *Nocardia*, *Brevibacterium*, and *Mycobacterium* (Itoh and Suzuki, 1974) and sucrose in the case of the same genera except *Mycobacterium* (Suzuki et al., 1974). Compounds for which structures have been elucidated are listed in Table 4.

**Table 4:** **Non-trehalose comprising glycolipids produced by actinobacteria**.

**Species**	**Strain**	**Hexose lipids**	**References**
*Arthrobacter paraffineus*	KY 4303	Sucrose mycolic (C32–C36)^*^	Suzuki et al., 1974
*Arthrobacter paraffineus*	KY 4303	Fructose coryne- and dicorynemycolic^*^	Itoh and Suzuki, 1974
*Arthrobacter* spp. *(globiformis, scleromae)*	ATCC 8010^T^ YH 2001^T^	Dimannosylacyl (C15–C17) monoglyceride (C15–C17) Galactosyl diglyceride(C15–C17)	Pasciak et al., 2010b
*Brevibacterium butanicum*	KY 4332	Fructose coryne- and dicorynemycolic^*^	Itoh and Suzuki, 1974
*Brevibacterium* spp.	n.a.	Sucrose mycolic (C32–C36)^*^	Suzuki et al., 1974
*Corynebacterium* spp.	n.a.	Sucrose mycolic (C32–C36)^*^	Suzuki et al., 1974
*Corynebacterium* spp.	n.a.	Fructose coryne- and dicorynemycolic^*^	Itoh and Suzuki, 1974
*Curtobacterium flaccumfaciens*	ATCC 13437	Di- and trimannosylglyceride (C18–C19 cyclopropane)	Mordarska et al., 1992
*Dietzia maris*	MCCC 1A00160	Rhamnolipid (C10/C10)	Wang et al., 2014
*Micrococcus lysodeikticus*	ATCC 4698	Dimannosylglyceride (C14)	Lennarz and Talamo, 1966
*Mycobacterium avium Mycobacterium koda*	KY 3844 KY 3852	Fructose coryne- and dicorynemycolic^*^	Itoh and Suzuki, 1974
*Nocardia butanica Nocardia convulutus*	KY 4333 KY 3907	Sucrose mycolic (C32–C36)^*^	Suzuki et al., 1974
*Nocardia rubra Nocardia butanica Nocardia convulutus*	KY 3844 KY 4333 KY 3907	Fructose coryne- and dicorynemycolic^*^	Itoh and Suzuki, 1974
*Nocardiopsis dassonvillei*	PCM 2492T (ATCC 23218)	Dimannosylacyl (C15) monoglyceride (C16)	Pasciak et al., 2004
*Rothia dentocariosa*	PCM 2249^T^ (ATCC 17931)	Dimannosylacyl monoglyceride (C16–C19)	Mordarska et al., 1992; Pasciak et al., 2002
*Rothia mucilaginosa*	PCM 2415^T^ (ATCC 25296^T^)	Dimannosylacyl (C15) monoglyceride (C16)	Pasciak et al., 2004
*Saccharopolyspora* spp. *(erythraea, hirsuta, rectivirgula*, sp.*)*	ATCC 27875^T^ ATCC 11635^T^ IMRU1258 LL-100-46)	Dimannosylacyl (C15–C16) monoglyceride (C16)	Gamian et al., 1996; Pasciak et al., 2002, 2004
*Sinomonas artrocyaneus*	LMG 3814^T^	Dimannoseylacyl (C14) monoglyceride (C16)	Niepel et al., 1997
**EXAMPLES OF NON-TREHALOSE COMPRISING HEXOSELIPIDS**			
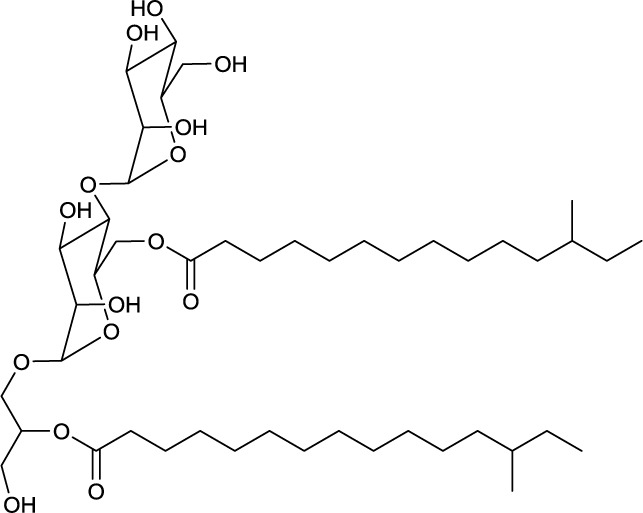		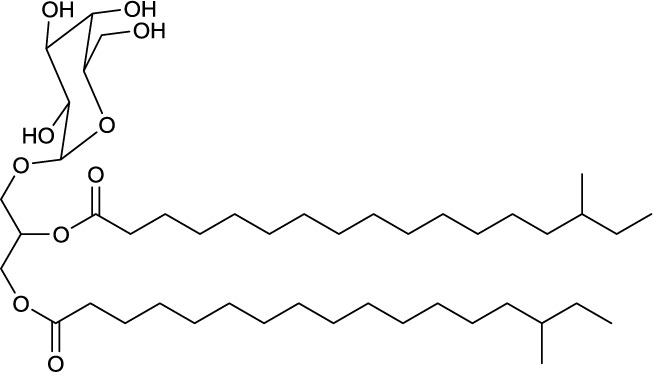	
**9**		**10**	
Dimannosylacyl monoglyceride produced by *Rothia mucilaginosa*		Galactosyl diglyceride produced by *Arthorbacter globiformis* and *Arthrobacter scleromae*	

*n.a., no information available*;

* *Sucrose and fructose based surfactants are variants of trehalose lipids*.

Besides the rhamnose-containing phenolic glycolipids mentioned in the oligosaccharide lipid section, the occurrence of other rhamnose-based lipids have recently been detected in a deep sea isolate identified as *Dietzia maris* (Wang et al., 2014) and has been identified as a C10:C10 di-rhamnolipid. This represents a unique occurrence within the class *Actinobacteria*. Other rhamnolipid producing actinobacteria are admittedly declared as producing strains in literature, however the surface active compounds produced have either not been elucidated or identified as rhamnolipids with debatable structural characterizations (*Rhodoccocus fascians* Gesheva et al., 2010, *Renibacterium salonarium* Christova et al., 2004, and a *Nocardioides* sp. Vasileva-Tonkova and Gesheva, 2005) (**Table 11**).

A different group of glycolipids are lipidic structures based on dimannose. Typically they are linked via a glycerol unit to different numbers of fatty acid chains. They have been reviewed in Shaw (1970) and structures have been identified for compounds produced by species belonging to the actinobacterial genera *Micrococcus* (Lennarz and Talamo, 1966), *Curtobacterium* (Mordarska et al., 1992), *Saccharopolyspora* (Gamian et al., 1996), *Rothia* (Pasciak et al., 2002, 2004), *Nocardiopsis* (Pasciak et al., 2004), *Arthrobacter* (Pasciak et al., 2010b) as well as the strain *Sinomonas atrocyaneus* (Niepel et al., 1997), formerly classified as *Arthrobacter atrocyaneus*. These di-mannose based glycolipids are composed of hydrophilic α-D-mannopyranose dimers linked with two C14–C16 *iso* or ante*iso* fatty acid chains. One chain is directly esterified to the C6 hydroxyl group of one sugar unit, while the second fatty acid chain is linked via a glycerol moiety to the C3 of the same sugar unit. The glycerol moiety is monoacylated at either the primary or secondary methylene position (**9**, Table 4) and its acylation site can be used to distinguish taxonomic properties of the different producer strains. These compounds have been isolated intracellularly and they act as precursors and cell membrane anchors for the synthesis of lipoarabinomannan, a polymeric surfactant and actinobacterial cell wall component (Pakkiri and Waechter, 2005) (see section on polymeric biosurfactants).

The coexistence of galactosyl diglycerides (**10**, Table 4) in *Arthrobacter scleromae* and *Arthrobacter globiformis* (Pasciak et al., 2010b) have been described and can be used as a glycomarker to distinguish these strains from the opportunistic pathogens, *Rothia mucilaginosa* and *Rothia dentocariosa* (Pasciak et al., 2002, 2004).

### Macrocyclic glycosides

Among the biosurfactants produced by actinobacteria, macrocyclic glycosides (Table 5) and macrocylcic dilactones (Table 6) can be distinguished and are often known to exhibit bio-activity against a range of organisms. The aliphatic macrolide antibiotic, brasilinolide, is produced by *Nocardia brasiliensis* and exhibits both antifungal and antibacterial activity. Three different variants have been described by Tanaka et al. (1997), Mikami et al. (2000) and Komatsu et al. (2004). All consist of a C32-membered macrolide with a sugar moiety but differ with regards to the acylation site of a malonic acid ester side chain (**11**, Table 5). The C16-membered dimeric macrolide elaiophylin and its variants have been isolated from various *Streptomyces* spp. including high producer strains. It exhibits bio-active properties against intestinal worms as well as antimicrobial, antitumor and immunosuppressant activities. A putative 95 kbp biosynthetic gene cluster of elaiophylin has been proposed (Haydock et al., 2004). Dembitsky (2005a,c) reviewed the different types of C14-membered lactam rings that are attached to an aminosugar (**12**, Table 5). Fluvirucin has been isolated from various *Actinomadura* spp., *Streptomyces* spp., *Microtetraspora* spp., and *Saccharotrix mutabilis*. The different fluvirucins share a common lactam ring unit but differ in terms of glycosylation. All of them act as potent antifungal agents against *Candida* spp. and show antiviral properties against influenza A virus (Dembitsky, 2005c).

**Table 5:** **Macrocyclic glycosides produced by actinobacteria**.

**Species**	**Strain**	**Macrocyclic glycoside**	**References**
*Actinomadura* spp.^*^ *(roseorufa roseorura, vulgaris, yumaensis)*	Various	Fluvirucin (14C macrolide)	Reviewed in: Dembitsky, 2005a,c
*Microtetraspora pusilla*	R359-5	Fluvirucin B1 (14C macrolide)	Dembitsky, 2005a
*Microtetraspora tyrrheni*	Q464-31	Fluvirucins (14C macrolide)	Dembitsky, 2005a,c
*Nocardia brasiliensis*	IFM 0406	Brasilinolide A, B, C (32C macrolide)	Tanaka et al., 1997; Mikami et al., 2000; Komatsu et al., 2004
*Saccharothrix mutabilis*	R869-9	Fluvirucin A2 (14C macrolide)	Dembitsky, 2005a
*Streptomyces* spp.^*^ (*antibioticus, erythreus, felleus, hygroscopicus, melanosporus, narbonensis, spinichromogenes, violaceoniger*)	various	Elaiophylin and derivates (16C macrolide) Fluvirucin (14C macrolide)	Reviewed in: Dembitsky, 2005a
**EXAMPLES OF MACROCYCLIC GLYCOSIDES**			
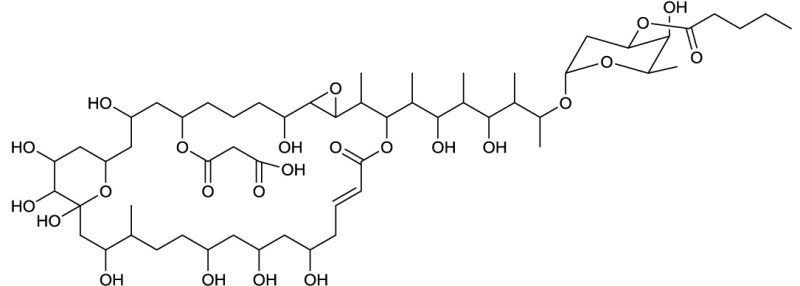		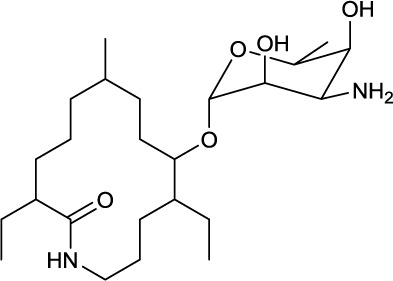	
**11**			**12**
Brasilinoide A produced by *Nocardia brasiliensis*		Fluvirucin B1 produced by *Actinomadura vulgaris* subsp. *lanata*	

* *Several producing strains are reported*.

**Table 6:** **Macrocyclic dilactones produced by actinobacteria**.

**Species**	**Strain**	**Macrocyclic dilactones**	**References**
*Kibdelosporangium albatum*	*ATCC 55061*	Cycloviracin B1 and B2 (C23/C26)	Tsunakawa et al., 1992a,b
*Nocardia vaccinii*	WC65712	Glucolypsin A and B (C19/C19)	Qian-Cutrone et al., 1999
*Streptomyces microflavus*	No.2445	Fattiviracin a1 (C22–28/C22–24)	Uyeda et al., 1998; Yokomizo et al., 1998
*Streptomyces purpurogeniscleroticus*	WC71634	Glucolypsin A and B (C19/C19)	Qian-Cutrone et al., 1999
**EXAMPLES OF MACROCYCLIC DILACTONES**			
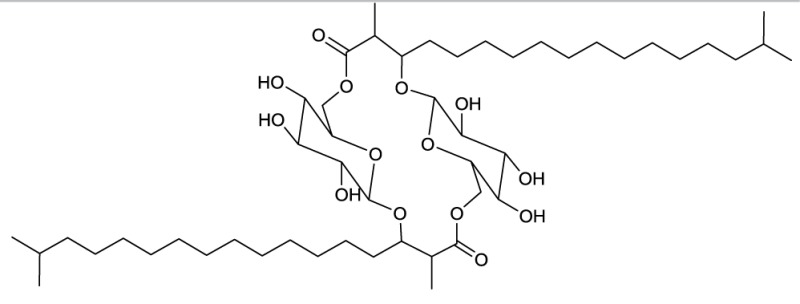			
**13**			
Glucolypsin A produced by *Nocardia vaccinii* and *Streptomyces purpurogeniscleroticus*			
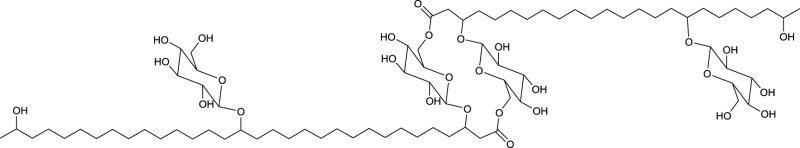			
**14**			
Fattiviracin produced by *Streptomyces microflavus*			
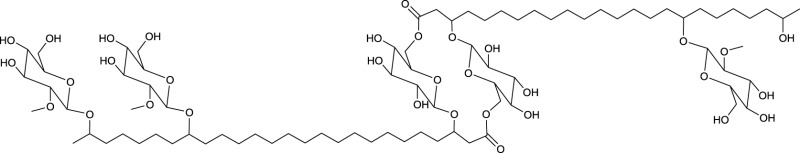			
**15**			
Cycloviracin B_1_ produced by *Kibdelosporangium albatum*			

Among the macrocyclic dilactones, glucolypsin, an acylglucose dimer has been isolated from *Streptomyces purpurogeniscleroticus* and *Nocardia vaccinii* by Qian-Cutrone et al. (1999). This extraordinary glycolipid is formed out of two glucose units linked to identical iso-branched C18 acyl chains that each carry a methyl group at C2 and a hydroxyl group at C3 of the acyl chain. By connecting the C6′ of the glucose molecule to the carboxy-C1 of the fatty acid chain, a rotationally symmetric dimer is formed (**13**, Table 6). Glucolypsin variants with C18 and C17 fatty acid chains of the same type also occur. Glycolypsin is reported to increase the activity of glucokinases by relieving its inhibition via long chain fatty acyl CoA esters (Qian-Cutrone et al., 1999). Derivates of glucolypsin that share a common backbone, have been shown to exhibit antiviral and antibiotic properties. In contrast to glucolypsin, the acylglucose dimer of fattiviracins (C24/C26) and cycloviracins (C24/C33) are built up out of trihydroxy fatty acids, each of them glycosidic linked to a further glucose unit at the third hydroxyl group. Cycloviracins are characterized by a fifth glucose unit bound to the C26 fatty acid chain, the three non-cyclic sugar units are methoxylated at C2, and the methyl branches at C2 of the fatty acid moieties are missing. Congeners of fattiviracin are divided into five families according to the length of their fatty acid moiety with each family showing similar antiviral activity against herpes, influenza and human immunodeficiency viruses (Uyeda, 2003). No alterations in the fatty acid chain length of cycloviracins have been reported. Fattiviracins (**14**, Table 6) have been shown to be produced by *Streptomyces microflavus* (Uyeda et al., 1998) and cycloviracins (**15**, Table 6) by *Kibdelosporangium albatum* (Tsunakawa et al., 1992b).

### Terpenoids and terpene glycosides

Actinobacterial terpenoid and terpene glycosides are summarized in Table 7. Vancoresmycin is a C65 highly oxygenated terpenoid glycoside produced by an *Amycolatopsis* sp. It contains a tetramic acid unit and is glycosidic linked to a methylated carbohydrate moiety containing one amino group (**16**, Table 7). Antimicrobial effect against various bacteria was reported by Hopmann et al. (2002), most notably against species resistant to the antibiotic vancomycin (often considered to be the antibiotic of last resort for the treatment of resistant bacteria). Besides the terpenoid glycoside, several different types of terpene glycosides are produced by actinobacterial strains. They are surfactants that mostly carry terminal hydrophilic groups linked by a hydrophobic carotenoid moiety.

**Table 7:** **Terpenoid and terpene-containing biosurfactants produced by actinobacteria**.

**Species**	**Strain**	**Terpenoids and terpenes**	**References**
*Amycolatopsis* sp.	DSM 12216	Vancoresmycin (65C terpenoid)	Hopmann et al., 2002
*Arthrobacter* sp.	M3	Corynexanthin mono- and diglycosides (C50 terpene)	Arpin et al., 1972; Dembitsky, 2005b
*Corynebacterium* sp.	CMB 8	Corynexanthin (C50 terpene)	Weeks and Andrewes, 1970
*Micrococcus yunnanensis*	AOY-1	Sarcinaxanthin, sarcinaxanthin mono- and diglucosides (C50 terpene)	Osawa et al., 2010
*Rhodococcus rhodochrous*	RNMS1	Carotenoid (C40 terpene) glycoside (C36–C50 mycolic)	Takaichi et al., 1997
**EXAMPLES OF TERPENE AND TERPENOID GLYCOSIDES**			
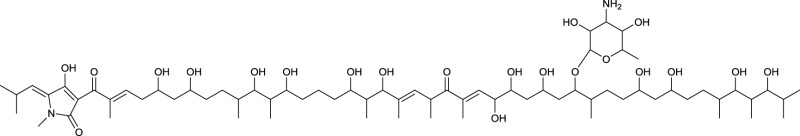			
**16**			
The terpenoidic glycoside vancoresmycin produced by *Amycolatopsis* sp.			
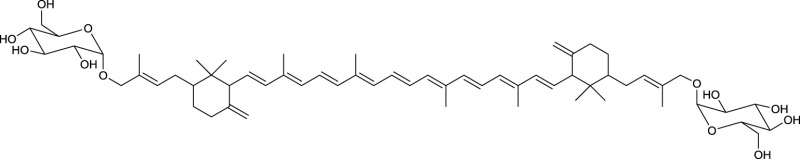			
**17**			
Sarcinaxanthin diglycoside produced by *Micrococcus yunnanensis*			
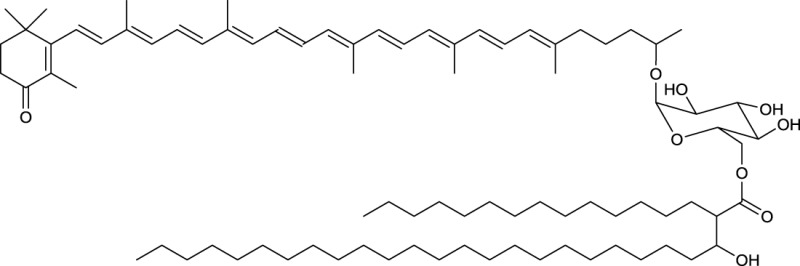			
**18**			
Carotenoid glycoside esterified with a rhodococcus type mycolic acid produced by *Rhodococcus rhodochrous*			

Terpene glycosides have been elucidated as products obtained from members of the following genera: *Corynebacterium* (Weeks and Andrewes, 1970)*, Arthrobacter* (Arpin et al., 1972), *Rhodococcus* (Takaichi et al., 1997), and *Micrococcus* (Osawa et al., 2010) (Table 7). Most of them share a backbone of a C50 atom carotenoid. They can either be linked to one or two hydroxyl groups at the terminal ends (decaprenoxanthin and sarcinaxanthin) or one hydroxyl group and one glycosidic moiety (corynexanthin, decaprenoxanthin monoglycoside and sarcinaxanthin monoglycoside). Di-glycosylated forms are found within *Arthrobacter* and *Micrococcus* (decaprenoxanthin diglucoside and sarcinaxanthin diglucoside) (**17**, Table 7) and further exist as an acetylated form at all hydroxyl groups. The terpene glycosides produced by *Rhodococcus rhodochrous*, differ from the one mentioned above, as they contain a monocyclic carotenoid backbone linked to a glucopyranosyl residue at the non-cyclic end (**18**, Table 7). The glucose unit is further acylated at C6 to a C36–C50 mycolic acid moiety leading to carotenoid glucoside mycolic acid esters. These terpene glycosides are mainly found in pigmented bacteria and it is hypothesized that they act as antioxidants to protect organisms from injuries caused by free radicals (Osawa et al., 2010).

## Polymeric biosurfactants

The most common polymeric surfactants produced by actinobacteria are macro-amphiphilic lipoglycans such as lipoarabinomannan and its precursors, lipomannan and phosphatidylinositol mannosides. In contrast to the core of the actinobacterial cell wall, arabinogalactan and peptidoglycan, these polymeric lipoglycans are non-covalently attached to the cell membrane although phosphatidylinositol mannides are structurally related to lipomannan and lipoarabinomannan anchor units. These polymeric glycolipids have been isolated from *Mycobacterium* spp., *Gordonia* spp., *Rhodococcus* spp., *Dietzia maris, Tsukamurella paurometabolus, Turicella otitidis*, and *Amycolatopsis sulphurea* (Table 8). Except for *A. sulphurea*, all of these strains belong to the suborder *Corynebacteridae* that are known to contain mycolic acids in their cell wall. It comprises the presence of mycolic acids and contain lipid rich cell envelope structures (Sutcliffe, 1997) forming an extremely robust and impermeable cell envelope (Berg et al., 2007). Lipoarabinomannans are well known to cause immunorepressive functions in diseases such as tuberculosis and leprosy that are caused by the pathogenic mycobacterial strains *M. tuberculosis* and *M. leprae*. However, non-pathogenic species have also been shown to produce lipoarabinomannans and are reported to have an opposite effect thus stimulating pro-inflammatory responses (Briken et al., 2004). The mannan core of lipoarabinomannan and the number of branching units is species dependent. Further differences in its structure is traced back to capping motifs present at the non-reducing termini of the arabinosyl side chains. Mannan caps are mainly present in pathogenic strains, whereas inositol phosphate caps are present in non-pathogenic mycobacteria (Briken et al., 2004). Lipoarabinomannans show structural similarity to its precursors lipomannan and phosphatidylinositol mannoside and consist of an α-1,6 linked mannan core with frequent α-1,2 mannose branches leading to a mannan backbone of approximately 20–25 mannose residues substituted with arabinofuran residues that carry terminal extension motifs, which vary among the producer species (Berg et al., 2007). The lipophilic part consists mainly of C16 glycerides that are linked to the mannan core by a phosphate group (**19**, Table 8).

**Table 8:** **Polymeric glycolipids of actinobacterial origin**.

**Species**	**Strain**	**Polymeric glycolipid**	**References**
*Corynebacterium matruchotii*	NCTC 10207	Lipoarabinomannan	Sutcliffe, 1995
*Turicella otitidis*	DSM 8821	Lipoarabinomannan	Gilleron et al., 2005
*Dietzia maris*	N1015	Lipoarabinomannan	Sutcliffe, 2000
*Mycobacterium* spp.^*^ (avium, bovis, chelonae, fortuitum, kansaii, leprae, smegmatis, tuberculosis, etc.)	Various	Lipoarabinomannan and lipomannan	Reviewed in: Chatterjee and Khoo, 1998; Brennan, 2003; Nigou et al., 2003; Briken et al., 2004
*Gordonia bronchialis*	N654^T^	Lipoarabinomannan, phosphatidylinositol mannoside	Garton and Sutcliffe, 2006
*Gordonia rubripertincta*	ATCC 25689	Lipoarabinomannan, phosphatidylinositol mannoside	Flaherty and Sutcliffe, 1999
*Rhodococcus* spp.^*^ (*equi, rhodnii, ruber*, etc)	Various	Lipoarabinomannan	Reviewed in: Sutcliffe, 1997
*Tsukamurella paurometabola*	DSM 20162	Lipoarabinomannan	Gibson et al., 2004
*Amycolatopsis sulphurea*	DSM 46092	Lipoarabinomannan	Gibson et al., 2003
**EXAMPLE OF LIPOARABINOMANNAN**			
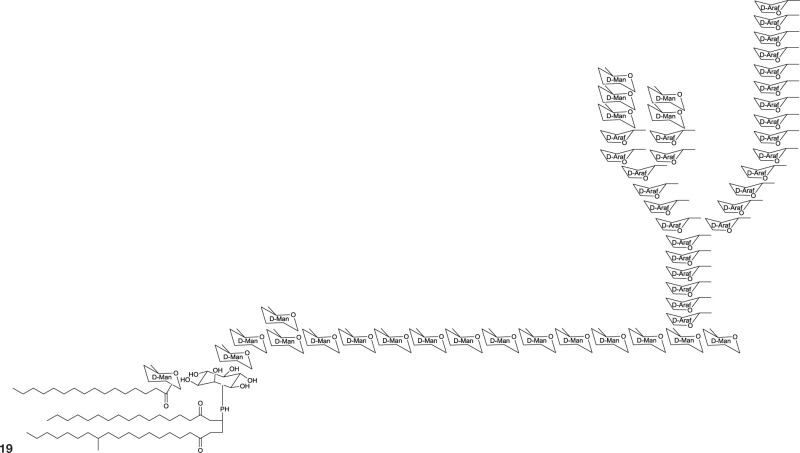			
Simplified structure of lipoarabinomannan produced by *Mycobacterium tuberculosis* with only one arabinofuran branch shown. Modified from Berg et al. (2007)			

* *Several producing strains are reported*.

## Lipopeptides

Cyclic and linear lipopeptides are produced by various actinobacterial strains and are summarized in Table 9.

**Table 9:** **Lipopeptides produced by actinobacterial strains**.

**Species**	**Strain**	**Lipopeptide**	**References**
*Actinoplanes* sp.	ATCC 33076	Ramoplanin (glycosylated 17aa, C8–C10)	Ciabatti et al., 1989; Gastaldo et al., 1992
*Kitasatospora cystarginea*	NRRL-B16505	Cystargamide (6aa, 2′-3′epoxy-C10)	Gill et al., 2014
*Rhodococcus* sp.	MCCC 1A00197	rhodocfactin	Peng et al., 2008
*Streptomyces roseosporus*	NRRL 11379	A21978C (daptomycin) (13aa, C10–12)	Debono et al., 1987
*Streptomyces tendae*	Tü 901/8c	Streptofactin	Richter et al., 1998
*Streptosporangium amethystogenes* subsp. *fukuiense*	AL-23456	TAN-1511 A, B, C	Takizawa et al., 1995
**EXAMPLES OF LIPOPEPTIDES**			
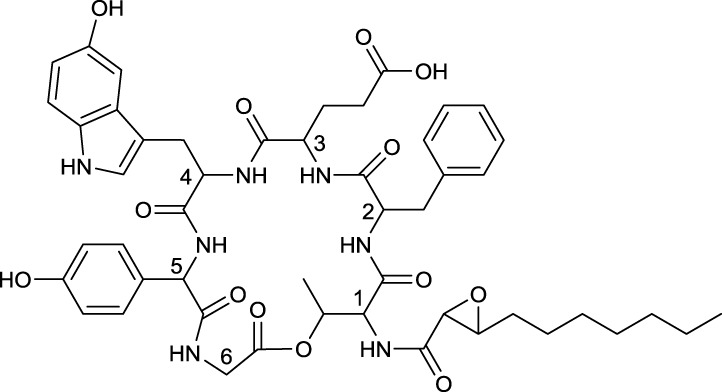		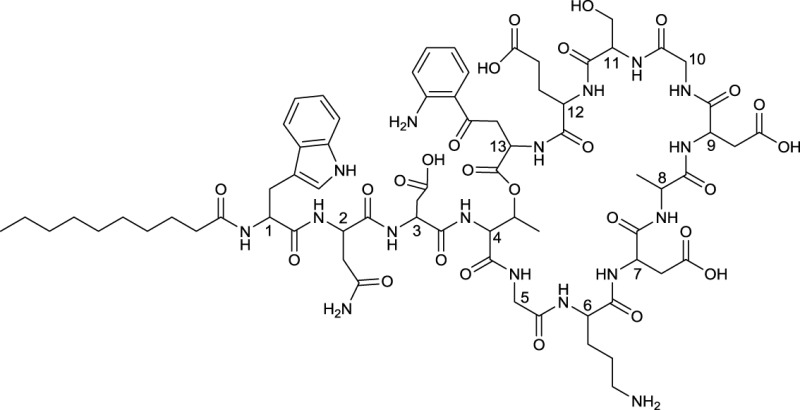	
**20**		**21**	
Cystargamide produced by *Kitasatospora cystarginea*		Daptomycin reacylated with decanoic acid from the core complex A21978C, produced by *Streptomyces roseasporus*	
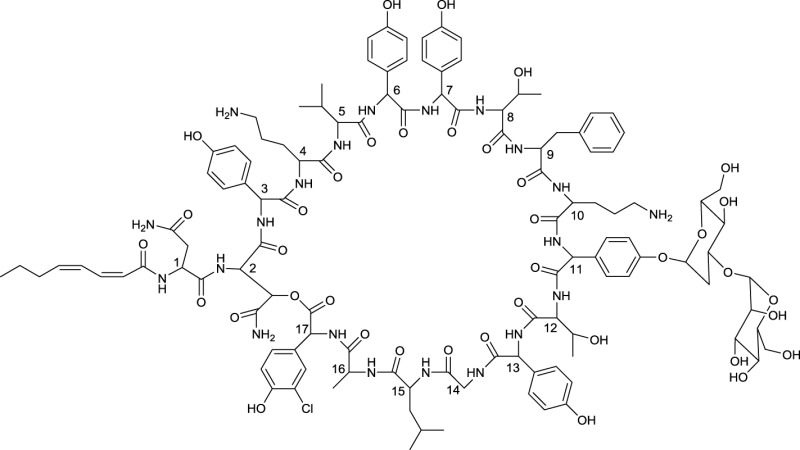			
**22**			
Dimannosylated ramoplanin produced by *Actinoplanes* sp.			
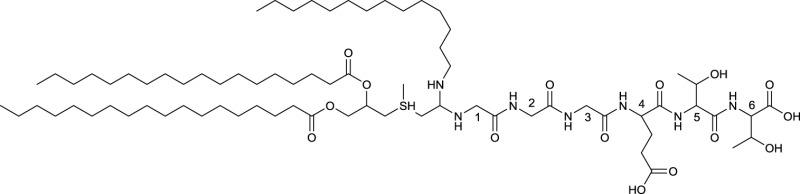			
**23**			
Linear TAN-1511 A produced by *Streptosporangium amethystogenes* subsp. *fukuiense*			

*aa, amino acid*.

### Cyclic lipopeptides

Cyclic lipopeptides are the most common type of lipopeptides and consist of a peptide chain of various types and numbers of amino acids circularized and linked to mainly one fatty acid chain. A surfactant often falsely cited to be produced by an actinobacterium but not of actinobacterial nature, is the eleven amino acid cyclic lipopeptide arthrofactin. It was initially postulated to be produced by an *Arthrobacter* sp. (Morikawa et al., 1993) but later corrected to originate from a *Pseudomonas* strain (Roongsawang et al., 2003).

Cyclic lipopeptides that have been reported within the class *Actinobacteria* are the six amino acid containing cystargamide produced by *Kitasatospora cystarginea* (Gill et al., 2014) (**20**, Table 9), the thirteen amino acid containing daptomycin produced by *Streptomyces roseosporus* (Debono et al., 1987) (**21**, Table 9) and the depsipeptide ramoplanin, containing 16 amino acids, and which is produced by an *Actinoplanes* sp. (Ciabatti et al., 1989) (**22**, Table 9). All of them are cyclic due to an ester linkage between the carboxyl terminus and a hydroxyl group of either a threonine or hydroxyl-asparagine.

In cystargamide, the smallest cyclic lipopeptide, an uncommon 2,3 epoxy fatty acid chain (C10) is linked to the threonine amine. Besides proteinogenic amino acids, cystargamide further contains rare 5′-hydroxy-trypthophan and 4′-hydroxyphenylglycine (**20**, Table 9). No antimicrobial activity of cystargamide could be demonstrated (Gill et al., 2014).

An outstanding example of successful screening for a surfactant with bioactive properties are A21978C complexes, known as precursors of daptomycin. They were structurally elucidated in 1987 (Debono et al., 1987) and A21978C comprises thirteen different amino acids, 10 of them in the cyclic part of the structure and three in the extension of the hydrophobic tail (**21**, Table 9). Three different lipophilic tails are known, C10 ante*iso*, C11 *iso* branched and C12 ante*iso*. The most bioactive form of A21978C is daptomycin and has been generated by enzymatic deacylation of the mixture of lipophilic tails and chemical reacylation with a decanoyl fatty acid moiety. It was approved by the U.S. Food and Drug Association (FDA) in 2003 as the first antibiotic of its kind, and commercialized as cubicin®. It is active against various gram positive bacteria including the methicilin-resistant pathogen *Staphylococcus aureus*, penicillin-resistant *Streptococcus pneumoniae* and vancomycin resistant enterococci (Miao et al., 2005). Its ability to act as an antimicrobial requires the presence of calcium. The cyclic lipopeptide oligomerizes and uses its C10 hydrophobic tail to interact with the bacterial membrane creating a membrane perforation and cell death. This displays a novel mode of action among antimicrobial agents. Daptomycin shows high activity and a resistance to its mechanism is more difficult to generate compared to conventional antibiotics (Vilhena and Bettencourt, 2012). It is produced by a non-ribosomal peptide synthetase (NRPS) in *S. roseosporus*. The NRPS contains three subunits whose main genes have recently been identified in a 128 kb cluster as *dpt*A, *dpt*BC, and *dpt*D (Miao et al., 2005) with several other genes necessary to synthesize an active form of daptomycin. Its production yield of approximately 0.5 g l^−1^, is relatively low compared to industrial production of other microbial products. Current attempts for a heterologous production not only target novel congeners of daptomycin but also the search for high producing strains. Similar production yields compared to the wild type strain have been reported for heterologous production which was developed using a combination of metabolic flux analysis and genetic modifications (Huang et al., 2012).

Antimicrobial activity against gram positive bacteria has also been detected for ramoplanin produced by an *Actinoplanes* sp. It contains 17 amino acids, 16 of which are part of the cyclic section of the compound. It is further glycosylated at a hydroxyphenylglycine with either di-mannose (Ciabatti et al., 1989) or mannose (Gastaldo et al., 1992), thus its classification as a glycolipopeptide. Besides its glycosylation pattern, members of ramoplanin can be differentiated by their acyl amides that consist of different di-unsaturated fatty acids linked to the distal hydroxyl-asparagine. The fatty acid chain varies in length between C8 and terminal branched C9 and C10 (**22**, Table 9).

A peptide-based surfactant produced by *Streptomyces tendae*, streptofactin, was found to contain hydrophobic amino acids, but lacked fatty acid chains (Richter et al., 1998).

### Linear lipopeptides

Linear lipopeptides have been found in *Streptosporangium amethystogenes* (Takizawa et al., 1995). They are reported to protect against infections in patients with leucopenia caused by cancer therapies by stimulating bone marrow cells. Different structures of these compounds are described, all share a 4′-thio C7 fatty acid chain with two ester linked C16–C19 fatty acid chains and one amide linked C13–C15 fatty acid chain. Three glycine amino acids are linked at the amide bond of the thio fatty acid with three to four proceeding amino acids varying in type (**23**, Table 9).

## Other actinobacterial biosurfactants

### Phenazine ester

Phenazines are a rare class of alkaloid esters. A marine *Streptomyces* sp. has been described to produce a phenazine ester that contain the desoxy pyranose quinovose esterified at either C3 or C4 to the carboxyl end of the phenazine. This phenazine-quinovose ester has been shown to exhibit antimicrobial activity. Several different types of the compound have been characterized also varying in hydroxylation and acetylation pattern at the desoxyglucose unit (Pathirana et al., 1992) (**24**, Table 10).

**Table 10:** **Other biosurfactants produced by actinobacteria**.

**Species**	**Strain**	**Compound**	**References**
*Streptomyces* sp.	CNB-253	Phenazine-quinovose	Pathirana et al., 1992
*Streptomyces* spp.^*^ (*griseoflavus, griseosporus, halstedii, lysosuperficus, nursei, vinausdrappus*)	various	Fatty acid amide glycoside (Tunicamycin, Streptovirudin, Liposidomycins)	Reviewed in: Dembitsky, 2005c
*Corynebacterium rathayi*	n.a.	Corynetoxin	Frahn et al., 1984
**EXAMPLES**			
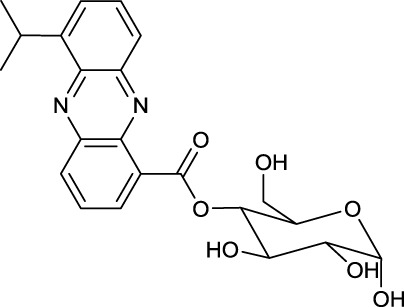		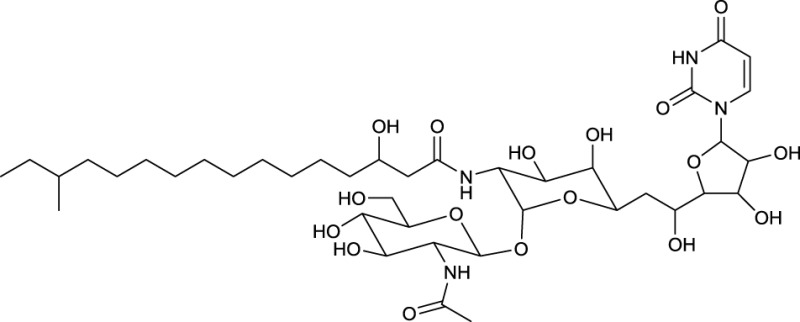	
**24**		**25**	
Phenanzine-quinovose ester produced by *Streptomyces* sp.		Corynetoxin produced by *Corynebacterium rathayi*	
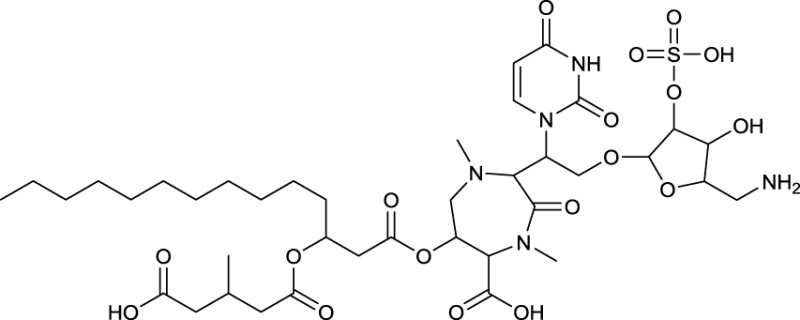			
**26**			
Liposidomycin A produced by *Streptomyces* sp.			

* *Several producing strains are reported*.

### Amide glycosides

Various surfactants with nucleoside fatty amide glycoside structure are produced by actinobacteria. A group of amide glucosides is based on the uracil and disaccharide-containing tunicamycin, a glycoprotein with antibacterial properties (Dembitsky, 2005c). In this glycoprotein, two saturated or unsaturated partly branched fatty acid chains varying in length are linked via an amide to the galactosamine/glucosamine disaccharide. Besides tunicamycin, produced by *Streptomyces* spp., the tunicamycin-based surfactants streptovirudin (containing dihydrouracil) and corynetoxin (**25**, Table 10) have been reported. The latter is produced by *Corynebacterium rathayi*, a pathogen of rye grass. The organism multiplies within the galls of sheep spreading the toxic metabolite (Frahn et al., 1984). In addition, the inhibitors of bacterial peptidoglycan synthesis, liposidomycin A, B, and C, have been reported to be produced by *Streptomyces griseosporus*. Liposidomycin A contains the so far uniquely described fatty acid composition of 3′-hydroxy-7,10-hexadecanoic acid (Dembitsky, 2005c) (**26**, Table 10).

## Not yet elucidated surfactants and their producing strains

Surface or emulsifying activity has been observed to occur from secondary metabolites of other members of the class *Actinobacteria*. Table 11 gives an overview of strains that are described to produce surface active compounds. Only some of the structures of these compounds have been partially elucidated.

**Table 11:** **Actinobacterial strains identified to produce surface active compounds for which no structures have been elucidated**.

**Species**	**Strain**	**Compound**	**References**
*Actinopolyspora* sp.	A18	n.d. GLP	Doshi et al., 2010
*Amycolatopsis tucumanensis*	DSM 45259	n.d. (bioemulsifier)	Colin et al., 2013
*Brachybacterium paraconglomeratum*	MSA21	n.d. GL (putative furan lipid/C12)	Kiran et al., 2014
*Brevibacterium aureum*	MSA13	n.d. LP (putative brevifactin/C18)	Kiran et al., 2010c
*Brevibacterium casei*	MSA19	n.d. GL (putative furan lipid/C18)	Kiran et al., 2010a
*Corynebacterium hydrocarboclastus*	n.a.	n.d. polymer	Zajic et al., 1977
*Corynebacterium lepus*	n.a.	n.d. LP	Cooper et al., 1979a
*Corynebacterium lepus*	n.a.	n.d. GL	Cooper et al., 1979a
*Corynebacterium lepus*	n.a.	p.d. (lipid, fatty acid, mycolic acid)	Cooper et al., 1979b
*Corynebacterium xerosis*	n.a.	n.d. LP	Margaritis et al., 1979
*Dietzia maris*	WR-3	p.d. (putative wax-ester)	Nakano et al., 2011
*Dietzia* sp.	S-JS-1	n.d. LP	Liu et al., 2009
*Frankia* sp.	CpI1	n.d. GL	Tunlid et al., 1989
*Gordonia amarae*	SC1	n.d. (extracellular with high molecular weight)	Iwahori et al., 2001
*Gordonia rubripertincta*	DSM 46038	n.d.	Pizzul et al., 2006
*Gordonia* sp.	ADP	n.d.	Pizzul et al., 2006
*Gordonia* sp.	BS29	n.d. GL	Franzetti et al., 2010
*Gordonia* sp.	JE-1058	n.d. (extracellular)	Saeki et al., 2009
*Kocuria marina*	BS-15	n.d. LP	Sarafin et al., 2014
*Leucobacter komagatae*	183	p.d. LP	Saimmai et al., 2012b
*Microlunatus* sp.	NA2	n.d.	Saimmai et al., 2012a
*Nocardia erythropolis*	ATCC 4277	n.d. GL, PL	Macdonald et al., 1981
*Nocardioides* sp.	A-8	n.d. GL (putative Rhamnolipid)	Vasileva-Tonkova and Gesheva, 2005
*Nocardiopsis alba*	MSA10	n.d. LP	Gandhimathi et al., 2009
*Nocardiopsis lucentensis*	MSA04	n.d. GL (putative furan lipid/C9)	Kiran et al., 2010b
*Oerskovia xanthineolytica*	CIP 104849	p.d. GL (hexose, pentose C10–C18)	Arino et al., 1998
*Pseudonocardia* sp.	BSNC30C	n.d.	Ruggeri et al., 2009
*Renibacterium salmoninarum*	27BN	n.d. GL (putative Rhamnolipid)	Christova et al., 2004
*Rhodococcus fascians*	A-3	n.d. GL (putative Rhamnolipid)	Gesheva et al., 2010
*Streptomyces* sp.	n.a.	n.d. GL	Khopade et al., 2011

*GL, Glycolipid; GLP, Glycolipiopeptide; LP, Lipopeptide; PL, Phospholipid; n.a., information not available; n.d., not determined; p.d., partly determined*.

Partly characterized surface active flocculants consisting of lipids, fatty acids and corynemycolic fatty acids of *Corynebacterium lepus* have been described by Cooper et al. (1979b). In addition, eleven different glycolipids that consist of hexoses and pentoses linked to diverse fatty acid moieties that vary in length of C10–C18 have also been described.

Besides *D. maris* (see glycolipid section), three other putative rhamnolipid-producing actinobacteria have been described. Vasileva-Tonkova and Gesheva (2005) and Gesheva et al. (2010) detected thin layer retention values equal to L-rhamnose after acid hydrolysis of a biosurfactant produced by a *Nocardioides* sp. and *Rhodococcus fascians*. The putative rhamnolipid was not further examined in terms of the hydrophilic moiety or fatty acid compositions. Christova et al. (2004) reported the production of rhamnolipid by *Renibacterium solmonarium* in comparison to commercial rhamnolipids in thin layer chromatography and infrared spectroscopy. The infrared spectra showed homologies to ester and carboxylic groups; thin layer chromatographic data were not shown in the study. In all cases the detection of rhamnolipids were putative and further structural analyses remains necessary for confirmation.

Other surface active compounds were only putatively classified based on the component analysis of the crude extract toward lipid, peptide and carbohydrate compositions. Based on this limited information, it was concluded that the production of either glycolipids or lipopeptides took place (Table 11).

Mass spectroscopic analysis greatly assisted to partly characterize the putative wax esters produced by *D. maris* (Nakano et al., 2011). In addition, Kiran et al. (2010a,b, 2014) described the production of furan-containing glycolipids in *Brachybacterium* spp., *Brevibacterium* spp., and *Nocardiopsis* spp. By analyzing hydrophilic and hydrophobic moieties after acid hydrolization, database comparison of gas chromatography-mass spectroscopic plots were used. ^1^H NMR evaluation of compounds from the two latter strains were described to approve the resulting structure, however relative data were not shown.

Similar results have been observed for surface active extracts with a majority of peptidic compounds in the hydrophilic part in *Brevibacterium aurum* (Kiran et al., 2010c) where fractions of the biosurfactant showed molecular weights of C9–C29 methyl esters and a mass that putatively confers to a proline-leucine-glycine-glycine amino acid chain. However, mass spectroscopic database comparisons remains putative. *Leucobacter komagate* is described to produce surfactin or a surfactin-like lipopeptide. This was concluded from mass spectroscopy, ^1^H NMR and infrared spectral data by Saimmai et al. (2012b), but the full elucidation of the structures could not be achieved.

The long list of non-elucidated actinobacterial surface active compounds underlines the extraordinary potential of finding novel biosurfactants in actinobacteria and displays the great need for structure elucidation to allow for a better understanding of the novelty and biodiversity of the compounds produced.

## Structural elucidations of actinobacterial surfactants

Various factors have been shown to influence the production, extraction, purification and structure elucidation of novel biosurfactants produced by actinobacterial strains. Due to their phenotypic growth characteristics, distinct membrane compositions and their function within the utilization of hydrocarbons, the surfactants produced are often membrane integrated, membrane associated, extracellular or a mixture of the above, and is always dependent on their particular function within the producing strains. Commonly the compounds produced exhibit antimicrobial properties, on the one hand proposing wide ranging applications, on the other resulting in opposing challenges during the production process. Special considerations are necessary when aiming for the extraction of the compound in an adequate amount and purity for structural elucidation as well as surfactant characterization. This section gives an overview of the most common techniques used to achieve successful structural elucidations.

### Detection

Novel surfactant producing strains can be detected through the use of screening assays that determine a surfactant's activity either from liquid culture (cell-free supernatant or culture broth) or from solid agar plates. Various detection methods have been described, but they mostly focus on changes observed in surface tension or the solubilization and emulsification of hydrocarbons. High throughput compatible assays can be distinct from more precise assays that need several milliliters of the compound to be tested. The latter often are also applied to characterize the activity of a purified biosurfactant. Good reviews on screening techniques have been summarized by Walter et al. (2010) and Satpute et al. (2010).

### Production

The manufacturing capacity of biosurfactants by a bacterial culture is limited. Wild type producing strains of the best described microbial surfactants, cultured with optimized process methods in suitable media and culture vessels reach production quantities of up to 422 g l^−1^ for sophorose lipids (Daniel et al., 1998), 112 g l^−1^ for rhamnose lipids (Giani et al., 1996), 110 g l^−1^ for spiculisporic acids (Tabuchi et al., 1977), 106 g l^−1^ for mannosylerythritol lipids (Morita et al., 2008) and 3,6 g l^−1^ for surfactin (Yeh et al., 2005). These are rare exceptions within the typical amounts produced by microorganisms, which usually do not exceed milligram amounts. The production level is strongly influenced by non-favorable growth and production conditions due to a lack of knowledge about the organism used and compound produced when initially screening for novel surfactants or novel producer strains.

With a few exceptions (Qian-Cutrone et al., 1999; Kügler et al., 2014), the average minimum volume for successful structure elucidation of an actinobacterial biosurfactant, is typically 20 l. Harvesting of the surfactants is type dependent and either whole cell broth (intracellular or membrane associated surfactants) or cell free supernatant is used as a starting point.

### Glycolipids

A typical method for the extraction of surfactants from culture broth or supernatant is the use of two phase extractions. In a first step, if appropriate, non-polar solvents (e.g., n-hexane) are used to remove residual hydrocarbons from the cultivation broth. If extraction is carried out from whole cell broth or wet cell mass, glycolipids are either captured by direct cell extraction or by cell treatment (e.g., sonication) prior to the extraction.

**Figure 2:**
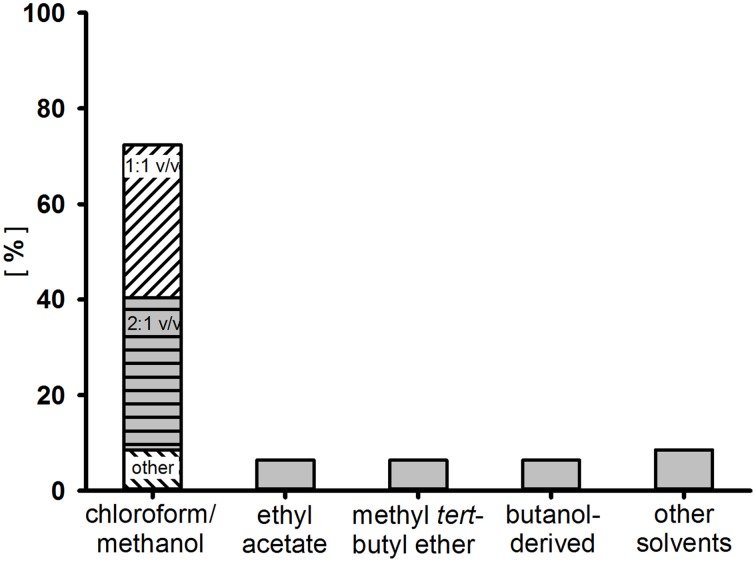
**Frequency distribution of solvents used for the enrichment of surfactants by two-phase extraction from the culture broth or cell free supernatant of 47 “rare” actinobacteria**.

In a second step, the surfactant is removed by repeated agitation with a medium polar solvent or solvent mixture. Most commonly, combinations of chloroform and methanol or polar aprotic solvents such as ethyl acetate or methyl-*tert*-butyl ether are used. A frequency solvent distribution for the extraction of glycolipids from “rare” actinobacteria is shown in Figure 2, comprising data of 47 two-phase extraction methods used to enrich surfactants produced from either cell-free supernatant or the culture broth. Depending on the chemical characteristics of the glycolipid, an acidification step (pH2–pH3) with subsequent incubation (4°C) prior to the extraction process could result in enhanced product recoveries (Passeri et al., 1990; Konishi et al., 2014). Often, after dehumification, further washing steps are applied, either of a hydrophilic (e.g., ultrapure water) or a hydrophobic (e.g., n-hexane) nature. For the polymeric glycolipid lipoarabinomannan and related structures, a hot-phenol water method is almost exclusively used (Sutcliffe, 2000).

The glycolipids produced, mainly present in mixtures of different forms, need to be separated for structural analysis. This procedure is usually performed by combinations of chromatographic steps using either gradient columns or preparative medium- and high pressure chromatography. In addition, preparative planar chromatographies are reported as an additional purification step for the isolation of pure compounds (Powalla et al., 1989; Pasciak et al., 2002, 2004). Rarely applied is the use of absorbers within the cultivation process. The number one choice for chromatography is the use of hydrophobicity affiliated separations with silicic acids as an absorbing material. In approximately 80% of structure reports from “rare” actinobacteria, silicic acid is used with various elution gradients of non-polar and polar solvents. Separated compounds are often further purified by repetitive silica chromatography using different gradients or by subsequent (or preceding) steps with different column material. Therefore, either reverse-phase C18 chromatography or cellulose-based ionic interaction chromatography are widely used.

### Lipopeptides

The diversity of different peptide-based surface active compounds produced by actinobacterial strains is much smaller than that of reported glycolipids. Depending on the lipopeptide produced, two different approaches for the concentration of the surfactants are used. Either the lipopeptide can be precipitated from the liquid culture/supernatant by either using cold acetone, methanol, salt concentrations, acidic environments, or a direct extraction by medium polar solvents similar to those used for glycolipids have been reported. Besides the chromatographic purification steps used for glycolipids, gel filtration has been successfully used as an additional step (Takizawa et al., 1995).

### Structural elucidation

Once a compound is purified to a sufficient extent, component analysis, specific staining methods and mass spectroscopic examinations are widely used to get a first hint about the type of surfactant produced. A more detailed schematic of the surfactant can be deduced from mass spectroscopy fragmentation studies, often revealing mass abundances of separated hydrophilic and hydrophobic parts of the glycolipid. However, complete structure examinations (of complete compounds or hydrolyzed components) rely on multi-dimensional nuclear magnetic resonance spectroscopy.

## Natural habitats of biosurfactant-producing actinobacteria

With the exception of a few strains, the great majority of surfactant-producing actinobacteria have been isolated from three different environments. These are: (1) Hydrocarbon contaminated soils, (2) infections caused by the actinobacterium itself, and (3) marine-derived samples. Obviously, this must not reflect the distribution of surfactant-producing actinobacteria in nature, but it is clear that there is a link between the type of environment and the ability of actinobacteria to produce biosurfactants and can be considered to be environmentally-driven.

### Hydrocarbon contaminated soil

The formation of various actinobacterial surfactants is mainly observed during growth in a range of different hydrophobic carbon sources such as n-paraffin, n-hexadecane or vegetable oils. Occurrences of surfactant-producing microorganisms seems to correlate to environments in which hydrophobic carbon sources are present, no matter if these are oil contaminated or oil enriched (Powalla et al., 1989; Arino et al., 1998; Christova et al., 2004, 2014; Pizzul et al., 2006; Liu et al., 2009; Ruggeri et al., 2009). Evoked by their hydrophobic cell wall due to incorporation and association of various lipoglycosides, actinobacteria preferably grow in hydrophobic droplets that are dispersed in the aqueous phase when cultured in cultivation devices. The surfactants produced facilitate the uptake of these difficult–to-access carbon sources by dispersing it into small droplets that can easily be pre-digested by extracellular enzymes.

### Infections

A second feature of surfactants is the antimicrobial property exhibited by most of these compounds. Endowed with nutritional and growth advantages toward surrounding organisms, surfactant producers can become rampant, and are often less affected by substances present during its growth, e.g., antimicrobial drugs. They have been found in patients that suffer from infections/diseases caused by human deficiency viruses (Guérardel et al., 2003), patients with lung infections and infections of the oral cavity (Datta and Takayama, 1993; Sutcliffe, 1995; Tanaka et al., 1997). In addition, biosurfactant-producing actinobacterial strains have also been isolated from infected plant tissue (Frahn et al., 1984).

### Marine habitat

Many actinobacteria are specialists in survival and native to a wide range of extreme environments. Surfactant-producing genera have been isolated from various marine-associated habitats (Passeri et al., 1990; Khopade et al., 2011; Nakano et al., 2011). Several of these environments exhibit rather extreme conditions, amongst which are deep sea sediments or hydrothermal fields (Peng et al., 2008; Konishi et al., 2014; Wang et al., 2014), ornithogenic exposed soil (Vasileva-Tonkova and Gesheva, 2005) as well as actinobacteria isolated from sponges (Gandhimathi et al., 2009; Kiran et al., 2010a,b,c, 2014) and hard corals (Osawa et al., 2010). An antimicrobial effect of surfactants produced in a highly procaryotic populated sponge tissue is apparent. However, the reason for the frequent occurrence of surfactant producers within the other marine habitats, still remains to be understood.

## Summary and conclusion

A wide range of unique and diverse surfactants produced by actinobacteria have been reported. Various glycolipids, lipopeptides and other surfactant types are produced by numerous species, all belonging to the order *Actinomycetales*. Taking into account the fact that only a minority of actinobacteria is culturable and the given list of surfactant producing strains without structurally elucidated compounds (Table 11), the sheer magnitude of actinobacterial surfactants that still remain undetermined is evident. The ability of actinobacteria to produce biosurfactants seems to be influenced by their natural habitat. From the three main sources of surfactant producing actinobacteria it can be concluded that the compounds produced mainly serve for either gaining access to hydrophobic carbon sources or as a bioactive agent against competing strains.

In order to pave the way toward biotechnological applications of actinobacterial surfactants, emphasis should be placed on (1) structural elucidation of described, but not identified biosurfactants, (2) the identification of novel actinobacterial surfactants by the implementation of next generation screening methods; (3) the production of sufficient amounts of surfactants for application based studies; and (4) production processes that result in high yields and that would cut down on the production costs.

1. Actinobacterial strains with a surface active culture broth or supernatant often are declared as “novel” biosurfactant producing strains, without elucidation of the surface active compound(s) produced and a list of producing strains is given in this article whose surfactant structures remain to be identified (Table 11). For a successful structural identification of the compound, sufficient quantities of the isolated surface active compound at an adequate purity is necessary in order to apply the various analytical methods necessary. This aspect was reviewed in the structural elucidation of actinobacterial compounds section. Quite a few of the studies cited lacked sufficient strain information and further research can only be ensured if the strains reported have designated strain numbers and thus are available for other researchers to pursue the production of these potentially novel biosurfactants.
2. Approaches for the identification of novel biosurfactants mainly remain traditional by the detection of interesting producing strains and subsequent isolation and characterization of the compound produced. To further expand the variety of actinobaterial surfactants, alternative screening methodologies that are already known to be used for the detection of novel lead molecules in the pharmaceutical industry could be applied. Genome-based information technology to reveal pathways that can be implemented into artificial surfactant synthesis cascades are currently being investigated. These attempts would allow for access to both undetected and cryptic pathways present in actinobacteria. By direct sequencing of metagenomic derived DNA, enzyme information acquired could be expanded to information gained from non-culturable and slow growing species.
3. Many of the surface active compounds produced by actinobacteria potentially show interesting properties as biotechnological products or additives. Often, as is the case for many of the compounds summarized in this article, an application based study is lacking. This is most probably due to low availability of the product and can be traced back to the use of low quantity producing strains. Focus on a novel actinobacterial surfactant, along with progress in the development toward novel biotechnology-based products, will only be made possible if enough substance for initial studies on bioactivity or other interesting applications can be acquired. If an adequate amount of substance is not achievable by standard bioprocess engineering attempts, metabolomic approaches and flux analysis could lead the way. Furthermore, the identification of enzymes involved in the synthesis and their genetic regulation can give an important input into the improvement of fermentation processes. An implementation of the surfactant's synthesis through adequate heterologous production strains could lead to higher quantities of the different surfactants produced. Potential applications of a novel compound is a guarantee of success in white biotechnology and negates the efforts made with regards to its production, purification and elucidation.
4. Currently, comparatively high production costs combined with low production yields restrict the development of compounds as valuable products, and are mainly limited to high purity applications, e.g., the drug industry. Several examples in the past have shown that once a potential application for a specific compound is foreseen, intensive research is set in motion to facilitate production and purification processes, cutting costs, enhancing yields and, although research often lasts for decades, compounds might end in industrial scale production and application.

One example of an actinobacterial surfactant that successfully underwent the process from detection to application is the antimicrobial agent daptomycin. It was initially produced semi-synthetically in a three step procedure, but later a direct synthesis of daptomycin was achieved by feeding toxic decanoic acid to a carbon-limited production culture (Huber et al., 1988). Production rates were further increased by 10–30% by using a mixture of less toxic decanal and a solvent to solubilize the hydrophobic carbon feed (Bertetti et al., 2012). Mutagenesis approaches (Yu et al., 2011; Li et al., 2013), genome shuffling (Yu et al., 2014) and directed overexpression (Huang et al., 2012), have recently led to further increases in production yields. Other examples of success stories, are non-actinobacterial surfactants that have been pushed to application: sophorolipids, mannosyl erythritol lipids and the lipopeptide surfactin have found application in cosmetic industries (Fracchia et al., 2014). Sophorolipids are even applied in low cost cleaning products.

Actinobacteria clearly represents a unique and vast untapped resource for the discovery of novel and potentially useful biosurfactants. The surfactants produced by members of the class *Actinobacteria* are a highly interesting group of products that could be of great importance in the future in both the area of basic research and application-oriented industrial research.

## Author contributions

JK has designed, conceived, and written this review, it's figures and tables as well as acquired and interpreted the relevant data used. All authors have fruitfully discussed content and structure of the review. In particular, ML has given substantial contributions related to actinobacteria and CS and RH have given substantial contributions related to biosurfactants.

### Conflict of interest statement

The authors declare that the research was conducted in the absence of any commercial or financial relationships that could be construed as a potential conflict of interest.

## Footnotes

^1^National Center for Biotechnology Information (NCBI) Taxonomy Browser. Available online at: http://www.ncbi.nlm.nih.gov/Taxonomy/Browser/wwwtax.cgi?mode=Undef&id=201174&lvl=5&lin (accessed 01.07.2014 - 07.01.2015).

## References

[B1] Abdelmohsen U. R. Bayer K. Hentschel U. (2014). Diversity, abundance and natural products of marine sponge-associated actinomycetes. Nat. Prod. Rep. 31, 381–399. 10.1039/c3np70111e 24496105

[B2] Arino S. Marchal R. Vandecasteele J.-P. (1998). Production of new extracellular glycolipids by a strain of *Cellulomonas cellulans* (*Oerskovia xanthineolytica*) and their structural characterization. Can. J. Microbiol. 44, 238–243 10.1139/w97-156

[B3] Arpin N. Liaaen-Jensen S. Trouilloud M. (1972). Bacterial carotenoids. XXXVIII. C 50-Carotenoids. 9. Isolation of decaprenoxanthin mono-and diglucoside from an *Arthrobacter* sp. Acta Chem. Scand. 26, 2524–2526. 4635696 10.3891/acta.chem.scand.26-2524

[B4] Asselineau C. Asselineau J. (1978). Trehalose-containing glycolipids. Prog. Chem. Fats Other Lipids 16, 59–99. 10.1016/0079-6832(78)90037-X 358271

[B5] Asselineau J. Lanéelle G. (1998). Mycobacterial lipids: a historical perspective. Front. Biosci. 3, e164–e174. 9751667 10.2741/a373

[B6] Azuma M. Suzutani T. Sazaki K. Yoshida I. Sakuma T. Yoshida T. (1987). Role of interferon in the augmented resistance of trehalose-6, 6′-dimycolate-treated mice to influenza virus infection. J. Gen. Virol. 68, 835–843. 10.1099/0022-1317-68-3-835 3493326

[B7] Baltz R. H. (2008). Renaissance in antibacterial discovery from actinomycetes. Curr. Opin. Pharmacol. 8, 557–563. 10.1016/j.coph.2008.04.008 18524678

[B8] Berdy J. (2005). Bioactive microbial metabolites. J. Antibiot. 58, 1–26. 10.1038/ja.2005.1 15813176

[B9] Bérdy J. (2012). Thoughts and facts about antibiotics: where we are now and where we are heading. J. Antibiot. 65, 385–395 10.1038/ja.2012.27 22511224

[B10] Berg S. Kaur D. Jackson M. Brennan P. J. (2007). The glycosyltransferases of *Mycobacterium tuberculosis*—roles in the synthesis of arabinogalactan, lipoarabinomannan, and other glycoconjugates. Glycobiology 17, 35R–56R. 10.1093/glycob/cwm010 17261566

[B11] Bertetti G. Malcangi A. Muraca R. Trione G. Rossi A. (2012). Process for the Production of Daptomycin. United states Patent 8, 313,922. Washington, DC: U.S. Patent and Trademark Office.

[B12] Brennan P. (2003). Structure, function, and biogenesis of the cell wall of *Mycobacterium tuberculosis*. Tuberculosis 83, 91–97. 10.1016/S1472-9792(02)00089-6 12758196

[B13] Brennan P. J. (1989). Structure of mycobacteria: recent developments in defining cell wall carbohydrates and proteins. Rev. Infec. Dis. 11, S420–S430. 10.1093/clinids/11.Supplement_2.S420 2469120

[B14] Brennan P. J. Lehane D. P. Thomas D. W. (1970). Acylglucoses of the Corynebacteria and Mycobacteria. Eur. J. Biochem. 13, 117–123. 10.1111/j.1432-1033.1970.tb00906.x 4314706

[B15] Briken V. Porcelli S. A. Besra G. S. Kremer L. (2004). Mycobacterial lipoarabinomannan and related lipoglycans: from biogenesis to modulation of the immune response. Mol. Microbiol. 53, 391–403. 10.1111/j.1365-2958.2004.04183.x 15228522

[B16] Chatterjee D. Khoo K.-H. (1998). Mycobacterial lipoarabinomannan: an extraordinary lipoheteroglycan with profound physiological effects. Glycobiology 8, 113–120. 10.1093/glycob/8.2.113 9451020

[B17] Christofi N. Ivshina I. (2002). Microbial surfactants and their use in field studies of soil remediation. J. Appl. Microbiol. 93, 915–929. 10.1046/j.1365-2672.2002.01774.x 12452947

[B18] Christova N. Lang S. Wray V. Kaloyanov K. Konstantinov S. Stoineva I. (2014). Production, structural elucidation and in vitro antitumor activity of trehalose lipid biosurfactant from Nocardia farcinica strain. J. Microbiol. Biotechnol. [Epub ahead of print]. 10.4014/jmb.1406.06025. 25370728

[B19] Christova N. Tuleva B. Lalchev Z. Jordanova A. Jordanov B. (2004). Rhamnolipid biosurfactants produced by *Renibacterium salmoninarum* 27BN during growth on n-hexadecane. Z. Naturforsch C 59, 70–74. 10.1515/znc-2004-1-215 15018056

[B20] Ciabatti R. Kettenring J. Winters G. Tuan G. Zerilli L. Cavalleri B. (1989). Ramoplanin (A-16686), a new glycolipodepsipeptide antibiotic. III. Structure elucidation. J. Antibiot. 42, 254–267. 10.7164/antibiotics.42.254 2597278

[B21] Colin V. L. Castro M. F. Amoroso M. J. Villegas L. B. (2013). Production of bioemulsifiers by *Amycolatopsis tucumanensis* DSM 45259 and their potential application in remediation technologies for soils contaminated with hexavalent chromium. J. Hazard. Mater. 261, 577–583. 10.1016/j.jhazmat.2013.08.005 23994656

[B22] Cooper D. G. Zajic J. E. Gerson D. F. (1979a). Production of surface-active lipids by *Corynebacterium lepus*. Appl. Environ. Microbiol. 37, 4–10. 760639 10.1128/aem.37.1.4-10.1979 PMC243394

[B23] Cooper D. G. Zajic J. E. Gracey D. (1979b). Analysis of corynomycolic acids and other fatty acids produced by *Corynebacterium lepus* grown on kerosene. J. Bacteriol. 137, 795–801. 422512 10.1128/jb.137.2.795-801.1979 PMC218359

[B24] Daniel H.-J. Reuss M. Syldatk C. (1998). Production of sophorolipids in high concentration from deproteinized whey and rapeseed oil in a two stage fed batch process using *Candida bombicola* ATCC 22214 and *Cryptococcus curvatus* ATCC 20509. Biotechnol. Lett. 20, 1153–1156 10.1023/A:1005332605003 10077820

[B25] Datta A. K. Takayama K. (1993). Isolation and purification of trehalose 6-mono-and 6,6′-di-corynomycolates from *Corynebacterium matruchotii*. Structural characterization by 1H NMR. Carbohyd. Res. 245, 151–158. 10.1016/0008-6215(93)80068-P 8358747

[B26] Debono M. Barnhart M. Carrell C. Hoffmann J. Occolowitz J. Abbott B.. (1987). A21978C, a complex of new acidic peptide antibiotics: isolation, chemistry, and mass spectral structure elucidation. J. Antibiot. 40, 761–777. 10.7164/antibiotics.40.761 3610833

[B27] Dembitsky V. M. (2004). Astonishing diversity of natural surfactants: 1. Glycosides of fatty acids and alcohols. Lipids 39, 933–953. 10.1007/s11745-004-1316-1 15691016

[B28] Dembitsky V. M. (2005a). Astonishing diversity of natural surfactants: 2. Polyether glycosidic ionophores and macrocyclic glycosides. Lipids 40, 219–248. 10.1007/s11745-005-1378-0 15957249

[B29] Dembitsky V. M. (2005b). Astonishing diversity of natural surfactants: 3. Carotenoid glycosides and isoprenoid glycolipids. Lipids 40, 535–557. 10.1007/s11745-005-1415-z 16149733

[B30] Dembitsky V. M. (2005c). Astonishing diversity of natural surfactants: 4. Fatty acid amide glycosides, their analogs and derivatives. Lipids 40, 641–660. 10.1007/s11745-005-1427-8 16196415

[B31] Domenech P. Reed M. B. Dowd C. S. Manca C. Kaplan G. Barry C. E. (2004). The role of MmpL8 in sulfatide biogenesis and virulence of *Mycobacterium tuberculosis*. J. Biol. Chem. 279, 21257–21265. 10.1074/jbc.M400324200 15001577

[B32] Doshi D. V. Maniyar J. P. Bhuyan S. S. Mujumdar S. S. (2010). Studies on bioemulsifier production by *Actinopolyspora* sp. A 18 isolated from garden soil. Indian J. Biotechnol. 9, 391–396 Available online at: http://nopr.niscair.res.in/handle/123456789/10437

[B33] Embley T. Stackebrandt E. (1994). The molecular phylogency and systematics of the actinomycetes. Annu. Rev. Microbiol. 48, 257–289. 10.1146/annurev.mi.48.100194.001353 7529976

[B35] Esch S. W. Morton M. D. Williams T. D. Buller C. S. (1999). A novel trisaccharide glycolipid biosurfactant containing trehalose bears ester-linked hexanoate, succinate, and acyloxyacyl moieties: NMR and MS characterization of the underivatized structure. Carbohyd. Res. 319, 112–123. 10.1016/S0008-6215(99)00122-6 10520259

[B36] Euzéby J. (2012). Validation List No. 144 List of new names and new combinations previously effectively, but not validly, published. Int. J. Syst. Evol. Microbiol. 62, 473–475 10.1099/ijs.0.041269-0 18319448

[B37] Euzéby J. (2013). Validation List No. 151 List of new names and new combinations previously effectively, but not validly, published. Int. J. Syst. Evol. Microbiol. 63, 1577–1580 10.1099/ijs.0.052571-0 18319448

[B38] Flaherty C. Sutcliffe I. C. (1999). Identification of a lipoarabinomannan-like lipoglycan in *Gordonia rubropertincta*. Syst. Appl. Microbiol. 22, 530–533. 10.1016/S0723-2020(99)80005-8 10794140

[B39] Fracchia L. Ceresa C. Franzetti A. Cavallo M. Gandolfi I. Van Hamme J. (2014). Industrial applications of biosurfactants. Biosurfactants: Production and Utilization—Processes, Technologies, and Economics, eds Kosaric N. Vardar-Sukan F. (Boca Raton; London; New York: CRC Press; Taylor & Francis Group), 159, 245 10.1201/b17599-15

[B40] Frahn J. Edgar J. Jones A. Cockrum P. Anderton N. Culvenor C. (1984). Structure of the corynetoxins, metabolites of *Corynebacterium rathayi* responsible for toxicity of annual ryegrass (*Lolium rigidum*) pastures. Aust. J. Chem. 37, 165–182 10.1071/CH9840165

[B41] Franzetti A. Gandolfi I. Bestetti G. Smyth T. J. Banat I. M. (2010). Production and applications of trehalose lipid biosurfactants. Eur. J. Lipid Sci. Technol. 112, 617–627. 10.1002/ejlt.20090016210068789

[B42] Gamian A. Mordarska H. Ekiel I. Ulrich J. Szponar B. A. Defaye J. (1996). Structural studies of the major glycolipid from *Saccharopolyspora* genus. Carbohyd. Res. 296, 55–67. 10.1016/S0008-6215(96)00246-7 9008843

[B43] Gandhimathi R. Kiran G. S. Hema T. A. Selvin J. Raviji T. R. Shanmughapriya S. (2009). Production and characterization of lipopeptide biosurfactant by a sponge-associated marine actinomycetes *Nocardiopsis alba* MSA10. Bioprocess Biosyst. Eng. 32, 825–835. 10.1007/s00449-009-0309-x 19288138

[B44] Garton N. J. Sutcliffe I. C. (2006). Identification of a lipoarabinomannan-like lipoglycan in the actinomycete *Gordonia bronchialis*. Arch. Microbiol. 184, 425–427. 10.1007/s00203-005-0050-z 16320036

[B45] Gastaldo L. Ciabatti R. Assi F. Restelli E. Kettenring J. K. Zerilli L. F.. (1992). Isolation, structure determination and biological activity of A-16686 factors A' 1, A' 2 and A' 3 glycolipodepsipeptide antibiotics. J. Ind. Microbiol. 11, 13–18. 10.1007/BF01583726 1369015

[B46] Gautier N. Marín L. M. L. Lanéelle M.-A. Daffé M. (1992). Structure of mycoside F, a family of trehalose-containing glycolipids of *Mycobacterium fortuitum*. FEMS Microbiol. Lett. 98, 81–87. 10.1111/j.1574-6968.1992.tb05494.x 1459422

[B47] Gesheva V. Stackebrandt E. Vasileva-Tonkova E. (2010). Biosurfactant production by halotolerant *Rhodococcus fascians* from Casey station, Wilkes land, Antarctica. Curr. Microbiol. 61, 112–117. 10.1007/s00284-010-9584-7 20135319

[B48] Giani C. Wullbrandt D. Rothert R. Meiwes J. (1996). Pseudomonas Aeruginosa and its Use in a Process for the Biotechnological Preparation of L-rhamnose. United states Patent 5,501,966. Washington, DC: U.S. Patent and Trademark Office.

[B49] Gibson K. J. Gilleron M. Constant P. Brando T. R. S. Puzo G. Besra G. S.. (2004). *Tsukamurella paurometabola* lipoglycan, a new lipoarabinomannan variant with pro-inflammatory activity. J. Biol. Chem. 279, 22973–22982. 10.1074/jbc.M310906200 15031299

[B50] Gibson K. J. Gilleron M. Constant P. Puzo G. Nigou J. Besra G. S. (2003). Identification of a novel mannose-capped lipoarabinomannan from *Amycolatopsis sulphurea*. Biochem. J. 372, 821–829. 10.1042/BJ20030197 12620092 PMC1223432

[B51] Gill K. A. Berrué F. Arens J. C. Kerr R. G. (2014). Isolation and structure elucidation of Cystargamide, a lipopeptide from *Kitasatospora cystarginea*. J. Nat. Prod. 77, 1372–1376. 10.1021/np500122s 24927492

[B52] Gilleron M. Garton N. J. Nigou J. R. M. Brando T. R. S. Puzo G. Sutcliffe I. C. (2005). Characterization of a truncated lipoarabinomannan from the Actinomycete *Turicella otitidis*. J. Bacteriol. 187, 854–861. 10.1128/JB.187.3.854-861.2005 15659663 PMC545729

[B53] Gilleron M. Stenger S. Mazorra Z. Wittke F. Mariotti S. Böhmer G.. (2004). Diacylated sulfoglycolipids are novel mycobacterial antigens stimulating CD1-restricted T cells during infection with *Mycobacterium tuberculosis*. J. Exp. Med. 199, 649–659. 10.1084/jem.20031097 14981115 PMC2213295

[B54] Goodfellow M. Fiedler H.-P. (2010). A guide to successful bioprospecting: informed by actinobacterial systematics. Antonie Van Leeuwenhoek 98, 119–142. 10.1007/s10482-010-9460-2 20582471

[B55] Goren M. B. (1970). Sulfolipid I of *Mycobacterium tuberculosis*, strain H37Rv II. Structural studies. Biochim. Biophys. Acta 210, 127–138. 10.1016/0005-2760(70)90068-8 4989542

[B56] Gudiña E. J. Rangarajan V. Sen R. Rodrigues L. R. (2013). Potential therapeutic applications of biosurfactants. Trends Pharmacol. Sci. 34, 667–675. 10.1016/j.tips.2013.10.002 24182625

[B57] Guérardel Y. Maes E. Briken V. Chirat F. Leroy Y. Locht C.. (2003). Lipomannan and lipoarabinomannan from a clinical isolate of *Mycobacterium kansasii* novel structural features and apoptosis-inducing properties. J. Biol. Chem. 278, 36637–36651. 10.1074/jbc.M305427200 12829695

[B58] Hausmann R. Syldatk C. (2014). Types and classification of microbial surfactants, in Biosurfactants: Production and Utilization—Processes, Technologies, and Economics, Vol. 159, eds Kosaric N. Vardar-Sukan F. (Boca Raton; London; New York: CRC Press; Taylor & Francis Group), 1.

[B59] Haydock S. F. Mironenko T. Ghoorahoo H. I. Leadlay P. F. (2004). The putative elaiophylin biosynthetic gene cluster in *Streptomyces* sp. DSM4137 is adjacent to genes encoding adenosylcobalamin-dependent methylmalonyl CoA mutase and to genes for synthesis of cobalamin. J. Biotechnol. 113, 55–68. 10.1016/j.jbiotec.2004.03.022 15380647

[B60] Henkel M. Müller M. M. Kügler J. H. Lovaglio R. B. Contiero J. Syldatk C.. (2012). Rhamnolipids as biosurfactants from renewable resources: Concepts for next-generation rhamnolipid production. Process Biochem. 47, 1207–1219. 10.1016/j.procbio.2012.04.01822728388

[B61] Hopmann C. Kurz M. Brönstrup M. Wink J. Lebeller D. (2002). Isolation and structure elucidation of vancoresmycin—a new antibiotic from *Amycolatopsis* sp. ST 101170. Tetrahedron. Lett. 43, 435–438 10.1016/S0040-4039(01)02171-2

[B62] Huang D. Wen J. Wang G. Yu G. Jia X. Chen Y. (2012). *In silico* aided metabolic engineering of *Streptomyces roseosporus* for daptomycin yield improvement. Appl. Microbiol. Biotechnol. 94, 637–649. 10.1007/s00253-011-3773-6 22406858

[B63] Huber F. Pieper R. Tietz A. (1988). The formation of daptomycin by supplying decanoic acid to *Streptomyces roseosporus* cultures producing the antibiotic complex A21978C. J. Biotechnol. 7, 283–292 10.1016/0168-1656(88)90040-5

[B64] Ishikawa E. Ishikawa T. Morita Y. S. Toyonaga K. Yamada H. Takeuchi O.. (2009). Direct recognition of the mycobacterial glycolipid, trehalose dimycolate, by C-type lectin Mincle. J. Exp. Med. 206, 2879–2888. 10.1084/jem.20091750 20008526 PMC2806462

[B65] Isoda H. Kitamoto D. Shinmoto H. Matsumura M. Nakahara T. (1997). Microbial extracellular glycolipid induction of differentiation and inhibition of protein kinase C activity of human promyelocytic leukaemia cell line HL60. Biosci. Biotechnol. Biochem. 61, 609–614. 10.1271/bbb.61.609 9145519

[B66] Itoh S. Suzuki T. (1974). Fructose-lipids of *Arthrobacter*, *Corynebacteria*, *Nocardia* and *Mycobacteria* grown on fructose. Agr. Biol. Chem. Tokyo 38, 1443–1449 10.1271/bbb1961.38.1443

[B67] Iwahori K. Tokutomi T. Miyata N. Fujita M. (2001). Formation of stable foam by the cells and culture supernatant of *Gordonia (Nocardia) amarae*. J. Biosci. Bioeng. 92, 77–79. 10.1016/S1389-1723(01)80203-6 16233062

[B68] Khan A. A. Stocker B. L. Timmer M. S. (2012). Trehalose glycolipids—synthesis and biological activities. Carbohyd. Res. 356, 25–36. 10.1016/j.carres.2012.03.010 22486827

[B69] Khopade A. Ren B. Liu X.-Y. Mahadik K. Zhang L. Kokare C. (2011). Production and characterization of biosurfactant from marine *Streptomyces* species B3. J. Colloid Interface Sci. 367, 311–318. 10.1016/j.jcis.2011.11.009 22138266

[B70] Kiran G. S. Sabarathnam B. Thajuddin N. Selvin J. (2014). Production of glycolipid biosurfactant from sponge-associated marine actinobacterium *Brachybacterium paraconglomeratum* MSA21. J. Surfactants Deterg. 17, 531–542 10.1007/s11743-014-1564-7

[B71] Kiran G. S. Sabu A. Selvin J. (2010a). Synthesis of silver nanoparticles by glycolipid biosurfactant produced from marine *Brevibacterium casei* MSA19. J. Biotechnol. 148, 221–225. 10.1016/j.jbiotec.2010.06.012 20600381

[B72] Kiran G. S. Thomas T. A. Selvin J. (2010b). Production of a new glycolipid biosurfactant from marine *Nocardiopsis lucentensis* MSA04 in solid-state cultivation. Colloids Surf. B Biointerfaces 78, 8–16. 10.1016/j.colsurfb.2010.01.028 20202801

[B73] Kiran G. S. Thomas T. A. Selvin J. Sabarathnam B. Lipton A. P. (2010c). Optimization and characterization of a new lipopeptide biosurfactant produced by marine *Brevibacterium aureum* MSA13 in solid state culture. Bioresour. Technol. 101, 2389–2396. 10.1016/j.biortech.2009.11.023 19959354

[B74] Komatsu K. Tsuda M. Tanaka Y. Mikami Y. Kobayashi J. I. (2004). Absolute stereochemistry of immunosuppressive macrolide brasilinolide A and its new congener brasilinolide C. J. Org. Chem. 69, 1535–1541. 10.1021/jo035773v 14987008

[B75] Konishi M. Nishi S. Fukuoka T. Kitamoto D. Watsuji T.-O. Nagano Y.. (2014). Deep-sea *Rhodococcus* sp. BS-15, lacking the phytopathogenic fas genes, produces a novel glucotriose lipid biosurfactant. Mar. Biotechnol. (NY) 16, 484–493. 10.1007/s10126-014-9568-x 24510374

[B76] Kügler J. H. Muhle-Goll C. Kühl B. Kraft A. Heinzler R. Kirschhöfer F.. (2014). Trehalose lipid biosurfactants produced by the actinomycetes *Tsukamurella spumae* and *T. pseudospumae*. Appl. Microbiol. Biotechnol. 98, 8905–8915. 10.1007/s00253-014-5972-4 25091045

[B77] Kurtboke I. (2010). Biodiscovery from microbial resources: actinomycetes leading the way. Microbiol. Aust. 31, 53–56 Available online at: http://microbiology.publish.csiro.au/?paper=MA10053

[B78] Kuyukina M. S. Ivshina I. B. (2010). Rhodococcus biosurfactants: biosynthesis, properties, and potential applications, in Biology of Rhodococcus ed Alvarez H. M. (Berlin, Heidelberg: Springer), 291–313.

[B79] Lanéelle M.-A. Asselineau J. (1977). Glycolipids of *Brevibacterium vitarumen*. Biochim. Biophys. Acta 486, 205–208. 10.1016/0005-2760(77)90085-6 1009134

[B80] Lang S. Philp J. C. (1998). Surface-active lipids in rhodococci. Antonie Van Leeuwenhoek 74, 59–70. 10.1023/A:1001799711799 10068789

[B81] Lennarz W. Talamo B. (1966). The chemical characterization and enzymatic synthesis of mannolipids in *Micrococcus lysodeikticus*. J. Biol. Chem. 241, 2707–2719. 5911642

[B82] Li L. Ma T. Liu Q. Huang Y. Hu C. Liao G. (2013). Improvement of daptomycin production in *Streptomyces roseosporus* through the acquisition of pleuromutilin resistance. Biomed. Res. Int. 2013:479742. 10.1155/2013/479742 24106707 PMC3782809

[B83] Liu J. Huang X.-F. Lu L.-J. Xu J.-C. Wen Y. Yang D.-H.. (2009). Comparison between waste frying oil and paraffin as carbon source in the production of biodemulsifier by *Dietzia* sp. S-JS-1. Bioresour. Technol. 100, 6481–6487. 10.1016/j.biortech.2009.07.006 19643603

[B84] Ludwig W. Klenk H.-P. (2001). Overview: a phylogenetic backbone and taxonomic framework for procaryotic systematics, in Bergey's Manual of Systematic Bacteriology, eds Boone D. R. Castenholz R. W. Garrity G. M. (New York, NY: Springer), 49–65.

[B85] Macdonald C. R. Cooper D. G. Zajic J. E. (1981). Surface-active lipids from *Nocardia erythropolis* grown on hydrocarbons. Appl. Environ. Microbiol. 41, 117–123. 16345679 10.1128/aem.41.1.117-123.1981 PMC243649

[B86] Manivasagan P. Venkatesan J. Sivakumar K. Kim S.-K. (2013). Marine actinobacterial metabolites: current status and future perspectives. Microbiol. Res. 168, 311–332. 10.1016/j.micres.2013.02.002 23480961

[B87] Marchant R. Banat I. M. (2012). Microbial biosurfactants: challenges and opportunities for future exploitation. Trends Biotechnol. 30, 558–565. 10.1016/j.tibtech.2012.07.003 22901730

[B88] Margaritis A. Zajic J. Gerson D. (1979). Production and surface-active properties of microbial surfactants. Biotechnol. Bioeng. 21, 1151–1162 10.1002/bit.260210706

[B89] Marques A. Pinazo A. Farfan M. Aranda F. Teruel J. Ortiz A.. (2009). The physicochemical properties and chemical composition of trehalose lipids produced by *Rhodococcus erythropolis* 51T7. Chem. Phys. Lipids 158, 110–117. 10.1016/j.chemphyslip.2009.01.001 19428355

[B90] Miao V. Coeffet-Legal M.-F. Brian P. Brost R. Penn J. Whiting A.. (2005). Daptomycin biosynthesis in *Streptomyces roseosporus*: cloning and analysis of the gene cluster and revision of peptide stereochemistry. Microbiology 151, 1507–1523. 10.1099/mic.0.27757-0 15870461

[B91] Mikami Y. Komaki H. Imai T. Yazawa K. Nemoto A. Tanaka Y.. (2000). A new antifungal macrolide component, brasilinolide B, produced by *Nocardia brasiliensis*. J. Antibiot. 53:70. 10.7164/antibiotics.53.70 10724012

[B92] Mordarska H. Gamian A. Ekiel I. (1992). Structural studies of major glycolipids from *Curtobacterium flaccumfaciens* pathovar *betae* and *Rothia dentocariosa*. Actinomycetes 3, 31–36.

[B93] Morikawa M. Daido H. Takao T. Murata S. Shimonishi Y. Imanaka T. (1993). A new lipopeptide biosurfactant produced by *Arthrobacter* sp. strain MIS38. J. Bacteriol. 175, 6459–6466. 8407822 10.1128/jb.175.20.6459-6466.1993 PMC206754

[B94] Morita T. Konishi M. Fukuoka T. Imura T. Sakai H. Kitamoto D. (2008). Efficient production of di- and tri-acylated mannosylerythritol lipids as glycolipid biosurfactants by *Pseudozyma parantarctica* JCM 11752^T^. J. Oleo Sci. 57, 557–565. 10.5650/jos.57.557 18781056

[B95] Müller M. M. Kügler J. H. Henkel M. Gerlitzki M. Hörmann B. Pöhnlein M.. (2012). Rhamnolipids—next generation surfactants? J. Biotechnol. 162, 366–380. 10.1016/j.jbiotec.2012.05.022 22728388

[B96] Nakano M. Kihara M. Iehata S. Tanaka R. Maeda H. Yoshikawa T. (2011). Wax ester-like compounds as biosurfactants produced by *Dietzia maris* from n-alkane as a sole carbon source. J. Basic Microbiol. 51, 490–498. 10.1002/jobm.201000420 21656811

[B97] Niepel T. Meyer H. Wray V. Abraham W.-R. (1997). A new type of glycolipid, 1-[α-mannopyranosyl-(1α-3)-(6-O-acyl-α-mannopyranosyl)]-3-O-acylglycerol, from *Arthrobacter atrocyaneus*. Tetrahedron 53, 3593–3602. 10.1016/S0040-4020(97)00079-310737205

[B98] Nigou J. R. M. Gilleron M. Puzo G. (2003). Lipoarabinomannans: from structure to biosynthesis. Biochimie 85, 153–166. 10.1016/S0300-9084(03)00048-8 12765785

[B99] Okazaki H. Sugino H. Kanzaki T. Fukuda H. Isobe K. (1969). l-glutamic acid fermentation: Part VI. structure of a sugar lipid produced by *Brevibacterium thiogenitalis* Part VII. Relation between biotin and oleic acid. Agr. Biol. Chem. Tokyo 33, 764–780 10.1271/bbb1961.33.764

[B100] Osawa A. Ishii Y. Sasamura N. Morita M. Kasai H. Maoka T. (2010). Characterization and antioxidative activities of rare C_50_ carotenoids-Sarcinaxanthin, Sarcinaxanthin monoglucoside, and Sarcinaxanthin diglucoside—obtained from *Micrococcus yunnanensis*. J. Oleo Sci. 59, 653–659 10.5650/jos.59.653 21099143

[B101] Pakkiri L. S. Waechter C. J. (2005). Dimannosyldiacylglycerol serves as a lipid anchor precursor in the assembly of the membrane-associated lipomannan in *Micrococcus luteus*. Glycobiology 15, 291–302. 10.1093/glycob/cwi003 15483271

[B102] Pasciak M. Ekiel I. Grzegorzewicz A. Mordarska H. Gamian A. (2002). Structure of the major glycolipid from *Rothia dentocariosa*. Biochim. Biophys. Act 1594, 199–205. 10.1016/S0167-4838(01)00301-6 11825622

[B103] Pasciak M. Holst O. Lindner B. Mierzchala M. Grzegorzewicz A. Mordarska H.. (2004). Structural and serological characterization of the major glycolipid from *Rothia mucilaginosa*. Biochim. Biophys. Act 1675, 54–61. 10.1016/j.bbagen.2004.08.004 15535967

[B104] Pasciak M. Kaczynski Z. Lindner B. Holst O. Gamian A. (2010a). Immunochemical studies of trehalose-containing major glycolipid from *Tsukamurella pulmonis*. Carbohyd. Res. 345, 1570–1574. 10.1016/j.carres.2010.04.026 20510396

[B105] Pasciak M. Sanchez-Carballo P. Duda-Madej A. Lindner B. Gamian A. Holst O. (2010b). Structural characterization of the major glycolipids from *Arthrobacter globiformis* and *Arthrobacter scleromae*. Carbohyd. Res. 345, 1497–1503. 10.1016/j.carres.2010.03.014 20381794

[B106] Passeri A. Lang S. Wagner F. Wray V. (1990). Marine biosurfactants, II. Production and characterization of an anionic trehalose tetraester from the marine bacterium *Arthrobacter* sp. EK 1. Z. Naturforsch 46, 204–209. 1878107 10.1515/znc-1991-3-408

[B107] Pathirana C. Jensen P. R. Dwight R. Fenical W. (1992). Rare phenazine L-quinovose esters from a marine actinomycete. J. Org. Chem. 57, 740–742 10.1021/jo00028a060

[B108] Peng F. Wang Y. Sun F. Liu Z. Lai Q. Shao Z. (2008). A novel lipopeptide produced by a Pacific Ocean deep-sea bacterium, *Rhodococcus* sp. TW53. J. Appl. Microbiol. 105, 698–705. 10.1111/j.1365-2672.2008.03816.x 18422956

[B109] Philp J. Kuyukina M. Ivshina I. Dunbar S. Christofi N. Lang S.. (2002). Alkanotrophic *Rhodococcus ruber* as a biosurfactant producer. Appl. Microbiol. Biotechnol. 59, 318–324. 10.1007/s00253-002-1018-4 12111164

[B110] Pizzul L. Pilar Castillo M. D. Stenström J. (2006). Characterization of selected actinomycetes degrading polyaromatic hydrocarbons in liquid culture and spiked soil. World J. Microbiol. Bioechnol. 22, 745–752 10.1007/s11274-005-9100-6

[B111] Powalla M. Lang S. Wray V. (1989). Penta-and disaccharide lipid formation by *Nocardia corynebacteroides* grown on n-alkanes. Appl. Microbiol. Biotechnol. 31, 473–479 10.1007/BF00270779

[B112] Qian-Cutrone J. Ueki T. Huang S. Mookhtiar K. A. Ezekiel R. Kalinowski S. S.. (1999). Glucolipsin A and B, two new glucokinase activators produced by *Streptomyces purpurogeniscleroticus* and *Nocardia vaccinii*. J. Antibiot. 52, 245–255. 10.7164/antibiotics.52.245 10348039

[B113] Richter M. Willey J. M. Süßmuth R. Jung G. Fiedler H.-P. (1998). Streptofactin, a novel biosurfactant with aerial mycelium inducing activity from *Streptomyces tendae* Tü 901/8c. FEMS Microbiol. Lett. 163, 165–171 10.1111/j.1574-6968.1998.tb13041.x

[B114] Ristau E. Wagner F. (1983). Formation of novel anionic trehalosetetraesters from *Rhodococcus erythropolis* under growth limiting conditions. Biotechnol. Lett. 5, 95–100 10.1007/BF00132166

[B115] Roongsawang N. Hase K.-I. Haruki M. Imanaka T. Morikawa M. Kanaya S. (2003). Cloning and characterization of the gene cluster encoding arthrofactin synthetase from *Pseudomonas* sp. MIS38. Chem. Biol. 10, 869–880. 10.1016/j.chembiol.2003.09.004 14522057

[B116] Ruggeri C. Franzetti A. Bestetti G. Caredda P. La Colla P. Pintus M. (2009). Isolation and characterisation of surface active compound-producing bacteria from hydrocarbon-contaminated environments. Int. Biodeterior. Biodegradation 63, 936–942 10.1016/j.ibiod.2009.05.003

[B117] Saadat S. Ballou C. (1983). Pyruvylated glycolipids from *Mycobacterium smegmatis*. Structures of two oligosaccharide components. J. Biol. Chem. 258, 1813–1818. 6822534

[B118] Saeki H. Sasaki M. Komatsu K. Miura A. Matsuda H. (2009). Oil spill remediation by using the remediation agent JE1058BS that contains a biosurfactant produced by *Gordonia* sp. strain JE-1058. Bioresour. Technol. 100, 572–577. 10.1016/j.biortech.2008.06.046 18692393

[B119] Saimmai A. Rukadee O. Onlamool T. Sobhon V. Maneerat S. (2012a). Characterization and phylogenetic analysis of microbial surface active compound-producing bacteria. Appl. Biochem. Biotechnol. 168, 1003–1018. 10.1007/s12010-012-9836-z 22899015

[B120] Saimmai A. Sobhon V. Maneerat S. (2012b). Production of biosurfactant from a new and promising strain of *Leucobacter komagatae* 183. Ann. Microbiol. 62, 391–402 10.1007/s13213-011-0275-9

[B121] Sarafin Y. Donio M. B. S. Velmurugan S. Michaelbabu M. Citarasu T. (2014). *Kocuria marina* BS-15 a biosurfactant producing halophilic bacteria isolated from solar salt works in India. Saudi J. Biol. Sci. 21, 511–519. 10.1016/j.sjbs.2014.01.001 25473358 PMC4250494

[B122] Satpute S. K. Banpurkar A. G. Dhakephalkar P. K. Banat I. M. Chopade B. A. (2010). Methods for investigating biosurfactants and bioemulsifiers: a review. Crit. Rev. Biotech. 30, 127–144. 10.3109/07388550903427280 20210700

[B123] Shao Z. (2011). Trehalolipids, in Biosurfactants—From Genes to Applications, ed Soberón-Chávez G. (Berlin; Heidelberg: Springer), 121–143.

[B124] Shaw N. (1970). Bacterial glycolipids. Bacteriol. Rev. 34, 365. 4924864 10.1128/br.34.4.365-377.1970 PMC378363

[B125] Singer M. V. Finnerty W. Tunelid A. (1990). Physical and chemical properties of a biosurfactant synthesized by *Rhodococcus* species H13-A. Can. J. Microbiol. 36, 746–750. 10.1139/m90-128 22049933

[B126] Sudo T. Zhao X. Wakamatsu Y. Shibahara M. Nomura N. Nakahara T.. (2000). Induction of the differentiation of human HL-60 promyelocytic leukemia cell line by succinoyl trehalose lipids. Cytotechnology 33, 259–264. 10.1023/A:1008137817944 19002834 PMC3466704

[B127] Sutcliffe I. C. (1995). Identification of a lipoarabinomannan-like lipoglycan in *Corynebacterium matruchotii*. Arch. Oral Biol. 40, 1119–1124. 10.1016/0003-9969(95)00086-0 8850650

[B128] Sutcliffe I. C. (1997). Macroamphiphilic cell envelope components of *Rhodococcus equi* and closely related bacteria. Vet. Microbiol. 56, 287–299. 10.1016/S0378-1135(97)00097-7 9226843

[B129] Sutcliffe I. C. (2000). Characterisation of a lipomannan lipoglycan from the mycolic acid containing actinomycete *Dietzia maris*. Antonie Van Leeuwenhoek 78, 195–201. 10.1023/A:1026562610490 11204771

[B130] Suzuki T. Tanaka H. Itoh S. (1974). Sucrose lipids of *Arthrobacteria*, *Corynebacteria* and *Nocardia* grown on sucrose. Agric. Biol. Chem. 38, 557–563 10.1271/bbb1961.38.557

[B131] Suzuki T. Tanaka K. Matsubara I. Kinoshita S. (1969). Trehalose lipid and α-branched-β-hydroxy fatty acid formed by bacteria grown on n-alkanes. Agr. Biol. Chem. Tokyo 33, 1619–1627 10.1271/bbb1961.33.1619

[B132] Tabuchi T. Nakamura I. Higashi E. Kobayashi H. (1977). Factors affecting the production of the open-ring acid of spiculisporic acid by *Penicillium spiculisporum*. J. Ferment Technol. 55, 43–49.

[B133] Takaichi S. Tamura Y. Azegami K. Yamamoto Y. Ishidsu J.-I. (1997). Carotenoid glucoside mycolic acid esters from the nocardioform actinomycetes, *Rhodococcus rhodochrous*. Phytochemistry 45, 505–508 10.1016/S0031-9422(97)00002-2

[B134] Takizawa M. Hida T. Horiguchi T. Hiramoto A. Harada S. Tanida S. (1995). TAN-1511 A, B and C, microbial lipopeptides with G-CSF and GM-CSF inducing activity. J. Antibiot. 48, 579–588. 10.7164/antibiotics.48.579 7544336

[B135] Tanaka Y. Komaki H. Yazawa K. Mikami Y. Nemoto A. Tojyo T.. (1997). Brasilinolide A, a new macrolide antibiotic produced by *Nocardia brasiliensis*: producing strain, isolation and biological activity. J. Antibiot. 50, 1036–1041. 10.7164/antibiotics.50.1036 9510911

[B136] Tokumoto Y. Nomura N. Uchiyama H. Imura T. Morita T. Fukuoka T.. (2009). Structural characterization and surface-active properties of a succinoyl trehalose lipid produced by *Rhodococcus* sp. SD-74. J. Oleo Sci. 58, 97–102. 10.5650/jos.58.97 19145064

[B137] Tsunakawa M. Komiyama N. Tenmyo O. Tomita K. Kawano K. Kotake C.. (1992a). New antiviral antibiotics, cycloviracins B1 and B2. I. Production, isolation, physico-chemical properties and biological activity. J. Antibiot. 45, 1467–1471. 10.7164/antibiotics.45.1467 1331014

[B138] Tsunakawa M. Kotake C. Yamasaki T. Moriyama T. Konishi M. Oki T. (1992b). New antiviral antibiotics, cycloviracins B1 and B2. II. Structure determination. J. Antibiot. 45, 1472–1480. 10.7164/antibiotics.45.1472 1429233

[B139] Tuleva B. Christova N. Cohen R. Antonova D. Todorov T. Stoineva I. (2009). Isolation and characterization of trehalose tetraester biosurfactants from a soil strain *Micrococcus luteus* BN56. Process Biochem. 44, 135–141 10.1016/j.procbio.2008.09.016

[B140] Tunlid A. Schultz N. A. Benson D. R. Steele D. B. White D. C. (1989). Differences in fatty acid composition between vegetative cells and N2-fixing vesicles of *Frankia* sp. strain CpI1. Proc. Natl. Acad. Sci. U.S.A. 86, 3399–3403. 10.1073/pnas.86.9.3399 16594036 PMC287140

[B141] Uyeda M. (2003). Fattiviracins, antiviral antibiotics produced by an actinomycete. Actinomycetologica 17, 57–66. 10.3209/saj.17_5715297717

[B142] Uyeda M. Yokomizo K. Miyamoto Y. Habib E. (1998). Fattiviracin A1, a novel antiherpetic agent produced by *Streptomyces microflavus* Strain No. 2445. I. Taxonomy, fermentation, isolation, physico-chemical properties and structure elucidation. J. Antibiot. 51, 823–828. 10.7164/antibiotics.51.823 9820232

[B143] Vasileva-Tonkova E. Gesheva V. (2005). Glycolipids produced by Antarctic *Nocardioides* sp. during growth on n-paraffin. Process Biochem. 40, 2387–2391 10.1016/j.procbio.2004.09.018

[B144] Vergne I. Daffé M. (1998). Interaction of mycobacterial glycolipids with host cells. Front. Biosci. 3, d865–d876. 9693156 10.2741/A330

[B145] Vilhena C. Bettencourt A. (2012). Daptomycin: a review of properties, clinical use, drug delivery and resistance. Mini Rev. Med. Chem. 12, 202–209. 10.2174/1389557511209030202 22356191

[B146] Vollbrecht E. Heckmann R. Wray V. Nimtz M. Lang S. (1998). Production and structure elucidation of di-and oligosaccharide lipids (biosurfactants) from *Tsukamurella* sp. nov. Appl. Microbiol. Biotechnol. 50, 530–537. 10.1007/s002530051330 9866171

[B147] Walter V. Syldatk C. Hausmann R. (2010). Screening Concepts for the Isolation of Biosurfactant Producing Microorganisms, in Biosurfactants. Advances in Experimental Medicine and Biology, Vol. 672, ed Sen R. (New York, NY: Springer), 1–13. 10.1007/978-1-4419-5979-9_1 20545270

[B148] Wang W. Shao Z. Cai B. (2014). Oil degradation and biosurfactant production by the deep sea bacterium *Dietzia maris* As-13-3. Front. Microbiol. 5:711. 10.3389/fmicb.2014.00711 25566224 PMC4267283

[B149] Watanabe R. Yoo Y. C. Hata K. Mitobe M. Koike Y. Nishizawa M.. (1999). Inhibitory effect of trehalose dimycolate (TDM) and its stereoisometric derivatives, trehalose dicorynomycolates (TDCMs), with low toxicity on lung metastasis of tumour cells in mice. Vaccine 17, 1484–1492. 10.1016/S0264-410X(98)00367-3 10195785

[B150] Weeks O. Andrewes A. (1970). Structure of the glycosidic carotenoid corynexanthin. Arch. Biochem. Biophys. 137, 284–286. 10.1016/0003-9861(70)90435-2 5435062

[B151] Yeh M.-S. Wei Y.-H. Chang J.-S. (2005). Enhanced production of Surfactin from *Bacillus subtilis* by addition of solid carriers. Biotechnol. Prog. 21, 1329–1334. 10.1021/bp050040c 16080719

[B152] Yokomizo K. Miyamoto Y. Nagao K. Kumagae E. Habib E. Suzuki K.. (1998). Fattiviracin A1, a novel antiviral agent produced by *Streptomyces microflavus* strain No. 2445. II. Biological properties. J. Antibiot. 51, 1035–1039. 10.7164/antibiotics.51.1035 9918397

[B153] Yu G. Hu Y. Hui M. Chen L. Wang L. Liu N.. (2014). Genome shuffling of *Streptomyces roseosporus* for improving daptomycin production. Appl. Biochem. Biotechnol. 172, 2661–2669. 10.1007/s12010-013-0687-z 24425298

[B154] Yu G. Jia X. Wen J. Lu W. Wang G. Caiyin Q.. (2011). Strain Improvement of *Streptomyces roseosporus* for daptomycin production by rational screening of He-Ne laser and NTG induced mutants and kinetic modeling. Appl. Biochem. Biotechnol. 163, 729–743. 10.1007/s12010-010-9078-x 20886375

[B155] Zajic J. E. Gignard H. Gerson D. F. (1977). Properties and biodegradation of a bioemulsifier from *Corynebacterium hydrocarboclastus*. Biotechnol. Bioeng. 19, 1303–1320. 10.1002/bit.260190905 19111

[B156] Zaragoza A. Aranda F. J. Espuny M. J. Teruel J. A. Marqués A. Manresa A.. (2009). Mechanism of membrane permeabilization by a bacterial trehalose lipid biosurfactant produced by *Rhodococcus* sp. Langmuir 25, 7892–7898. 10.1021/la900480q 19391573

[B157] Zhi X.-Y. Li W.-J. Stackebrandt E. (2009). An update of the structure and 16S rRNA gene sequence-based definition of higher ranks of the class *Actinobacteria*, with the proposal of two new suborders and four new families and emended descriptions of the existing higher taxa. Int. J. Syst. Evol. Microbiol. 59, 589–608. 10.1099/ijs.0.65780-0 19244447

